# Xanthohumol ameliorates Diet-Induced Liver Dysfunction via Farnesoid X Receptor-Dependent and Independent Signaling

**DOI:** 10.3389/fphar.2021.643857

**Published:** 2021-04-20

**Authors:** Ines L. Paraiso, Thai Q. Tran, Armando Alcazar Magana, Payel Kundu, Jaewoo Choi, Claudia S. Maier, Gerd Bobe, Jacob Raber, Chrissa Kioussi, Jan F. Stevens

**Affiliations:** ^1^ Linus Pauling Institute, Oregon State University, Corvallis, OR, United States; ^2^ Department of Pharmaceutical Sciences, Oregon State University, Corvallis, OR, United States; ^3^ Department of Chemistry, Oregon State University, Corvallis, OR, United States; ^4^ Department of Behavioral Neuroscience, Oregon Health and Science University, Portland, OR, United States; ^5^ Department of Animal and Rangeland Sciences, Oregon State University, Corvallis, OR, United States; ^6^ Department of Neurology, Psychiatry and Radiation Medicine, Division of Neuroscience, Oregon National Primate Research Center, Oregon Health and Science University, Portland, OR, United States

**Keywords:** nonalcoholic fatty liver disease, farnesoid X receptor, bile acids, lipid metabolism, xanthohumol

## Abstract

The farnesoid X receptor (FXR) plays a critical role in the regulation of lipid and bile acid (BA) homeostasis. Hepatic FXR loss results in lipid and BA accumulation, and progression from hepatic steatosis to nonalcoholic steatohepatitis (NASH). This study aimed to evaluate the effects of xanthohumol (XN), a hop-derived compound mitigating metabolic syndrome, on liver damage induced by diet and FXR deficiency in mice. Wild-type (WT) and liver-specific FXR-null mice (FXR^Liver−/−^) were fed a high-fat diet (HFD) containing XN or the vehicle formation followed by histological characterization, lipid, BA and gene profiling. HFD supplemented with XN resulted in amelioration of hepatic steatosis and decreased BA concentrations in FXR^Liver−/−^ mice, the effect being stronger in male mice. XN induced the constitutive androstane receptor (CAR), pregnane X receptor (PXR) and glucocorticoid receptor (GR) gene expression in the liver of FXR^Liver−/−^ mice. These findings suggest that activation of BA detoxification pathways represents the predominant mechanism for controlling hydrophobic BA concentrations in FXR^Liver−/−^ mice. Collectively, these data indicated sex-dependent relationship between FXR, lipids and BAs, and suggest that XN ameliorates HFD-induced liver dysfunction via FXR-dependent and independent signaling.

## Introduction

Dyslipidemia coincides with other metabolic disorders such as obesity, hypertension, and glucose intolerance, defined as metabolic syndrome (MetS), which increase the risk to develop type 2 diabetes (T2D) and cardiovascular diseases (Porez et al., 2012). Obesity and T2D are also associated with nonalcoholic fatty liver disease (NAFLD), a spectrum of chronic liver abnormalities from simple steatosis to nonalcoholic steatohepatitis (NASH) to liver cirrhosis (Larter et al., 2010; Chiang, 2013). The growing prevalence of obesity and high-fat diet (HFD)-induced dyslipidemia represent a public health problem worldwide and the development of drugs with a combined effect on different risk factors may be more effective than the use of combinatorial therapy to manage patients' global risks.

Before the discovery of statins, hypercholesterolemia was primarily treated with bile acid (BA) sequestrants, which bind BAs in the intestine and prevent their reabsorption, thereby promoting the hepatic synthesis of BAs from cholesterol (Staels and Kuipers, 2007; Porez et al., 2012). BA synthesis in hepatocytes occurs largely through the classical pathway initiated by the rate-limiting enzyme cholesterol 7α-hydroxylase (CYP7A1). The classical pathway forms the primary BAs, cholic acid (CA) and chenodeoxycholic acid (CDCA), following a multistep enzymatic process. In complement, CYP27A1 initiates an alternative pathway of BA synthesis that also leads to CDCA synthesis (Garcia et al., 2018). Shortly after their synthesis, BAs are conjugated to glycine or taurine and stored into the gallbladder (Chiang, 2013; Garcia et al., 2018). Besides their involvement in transcriptional regulation of cholesterol metabolism (Chiang, 2002; Trauner et al., 2010), BAs regulate hepatic gluconeogenesis, glycogen synthesis and insulin sensitivity (Ma et al., 2006; Trauner et al., 2010). BAs also modulate neurotransmission, neuroendocrine responses, and neurogenesis indicating their importance in neurological functions (Schubring et al., 2012; McMillin and DeMorrow, 2016). However, BA accumulation causes inflammation, hepatic injury (Chiang, 2017) and is associated with motor and cognitive impairments (Huang et al., 2016; McMillin et al., 2016). A key regulator of maintaining lipid and BA homeostasis is the farnesoid X receptor (FXR, NR1H4), which upon activation by BAs, polyunsaturated fatty acids and farnesylated proteins (Forman et al., 1995; Makishima et al., 1999; Zhao et al., 2004), regulates the expression of target genes involved in various physiological processes (Chawla et al., 2001; Sun et al., 2021). An increase of intracellular BAs also activates the constitutive androstane receptor (CAR) and pregnane X receptor (PXR). They modulate transcriptional regulation of their targets including genes encoding hepatic BA metabolizing enzymes and BA/organic anion transporters (Guo et al., 2003; Uppal et al., 2005; Lee et al., 2006). Subsequently, FXR, CAR and PXR have emerged as promising targets for the treatment of metabolic disorders associated with MetS (Gao and Xie, 2012; Porez et al., 2012).

Xanthohumol (XN) is a hop-derived flavonoid, which mitigates obesity-related metabolic impairments by improving dysfunctional glucose and lipid metabolism in HFD-fed animals (Miranda et al., 2016; Miranda et al., 2018). Treatment of HFD-fed C57BL/6J mice with a diet containing XN decreases their plasma low-density lipoprotein cholesterol (LDL-c), IL-6, Homeostatic Model Assessment of Insulin Resistance (HOMA-IR) and leptin concentrations (Miranda et al., 2016). XN enhances fatty acid oxidation as a result of mild mitochondrial uncoupling (Kirkwood et al., 2013) and decreases adipocyte markers such as PPARγ, C/EBPα and DGAT1 (Yang et al., 2007). This effect might be at least partly mediated by FXR, since XN is a ligand of FXR (Yang et al., 2016) that modulates FXR downstream gene expression in a manner similar to selective bile acid receptor modulators (SBARM) (Nozawa, 2005; Paraiso et al., 2020). However, the extent to which FXR signaling mediates the *in vivo* effects of XN is unknown. Both activation of hepatic FXR and inhibition of intestinal FXR have beneficial effects in obesity-related metabolic diseases (Sun et al., 2021) due to differential effects on metabolic regulation (Kim et al., 2007; Schmitt et al., 2015). These effects are further emphasized by the observation that intestine-specific FXR knockout mice are resistant to HFD-induced obesity, while HFD-fed liver-specific FXR knockout mice develop NAFLD (Li et al., 2013; Schmitt et al., 2015). Therefore, tissue-specific mouse models are necessary to dissect the complex effects of FXR on dyslipidemia. In the current study, we used liver-specific FXR-null mice (FXR^Liver−/−^) to investigate the effect of XN on dyslipidemia and BA accumulation. Our findings demonstrate that XN ameliorate HFD-induced hepatic injury and dysfunctional lipid and BA metabolism in WT and FXR^Liver−/−^ mice. We also provide evidence that XN induces expression of nuclear receptors (NRs) including CAR, PXR and the glucocorticoid receptor (GR) involved in the metabolism of BAs and lipids. These findings have potentially important implications in the treatment of metabolic and cholestatic diseases.

## Materials and Methods

### Animal Studies

All animal experiments were performed in accordance with institutional and National Health and Medical Research Council guidelines. The experimental protocol was approved by the Institutional Animal Care and Use Committee at Oregon State University and the studies were carried out in accordance with the approved protocol (IACUC 2019-0001). Nine-week-old WT male and female C57BL/6J mice were obtained from Jackson Laboratory (Bar Harbor, ME, United States). FXR^Liver−/−^ mice were generated by crossing FXR^FL/FL^ mice with mice harboring the Cre recombinase under the control of the albumin promoter (Alb^Cre^) to produce the Alb^Cre^:FXR^FL/FL^ or FXR^Liver−/−^ mice (Kong et al., 2016). All mice were in C57BL/6J genetic background for over 12 generations. Mice were housed in groups of two–3 in ventilated cages under a 12–12-h light-dark cycle and fed a HFD (Dyets Inc. Bethlehem, PA, United States) containing 60, 20 and 20% total calories from fat, carbohydrate and protein, respectively. XN (purity >99%) from Hopsteiner Inc (New York, NY, United States) was mixed into the diet as previously described (Miranda et al., 2018) to deliver a dose of 60 mg/kg body weight/day. The control diet contained an identical amount of the vehicle. 15 WT mice (8 females, 7 males) and 18 FXR^Liver−/−^ mice (10 females, 8 males) were fed a control HFD, while 15 WT mice and 18 FXR^Liver−/−^ mice were treated with XN for a duration of 12 weeks. Food intake and body weights were recorded weekly. At week 10, fasting glucose was measured after 6 h of fasting by using the One Touch UltraMini glucometer (LifeScan Inc. Milpitas, CA, United States). At the end of 12 weeks of feeding, fed-state mice were euthanized by cervical dislocation, their blood collected, and their liver and hippocampus were dissected for further analyses. Deletion of FXR in the liver of FXR^Liver−/−^ mice was confirmed by genotyping at weaning (Kong et al., 2016). Quantitative PCR after the feeding experiment. FXR mRNA levels were ∼ 3-fold lower in the liver of mutant compared to WT mice (Supplementary Figure S1).

### Histology

Liver biopsies from *n* = 3 male mice per genotype-diet group were fixed in 4% paraformaldehyde, embedded in OCT and 10 µm-thick sections were used for histology. Hematoxylin and Eosin (H&E) and Sudan black staining were performed as previously described (Chang et al., 2019).

### Measurement of Hepatic Transaminase Activities and Plasma Leptin Concentrations

To measure ALT and AST enzymatic activities, liver samples (*n* = 6 per genotype-diet group) were homogenized in 10 ml of 100 mM Tris (pH = 7.8) per Gram of tissue. The homogenates were centrifuged at 10,000 ×g for 15 min at 4°C. The supernatants were analyzed for ALT and AST activity using colorimetric assay kits purchased from Cayman Chemical (Ann Arbor, MI, United States). Plasma leptin concentrations (n = 5–7 per genotype-diet group) were measured using the Enzyme Immunoassay kit from SPI Bio Inc.. (Sherbrooke, QC, Canada) as per manufacturer’s instructions.

### Liver Lipidomics

Mouse liver samples (50 mg, n = 15–18 per genotype-diet group) were spiked with SPLASH^®^ Lipidomix^®^ internal standards from Avanti Lipids (Alabaster, AL, United States) and homogenized with zirconium oxide beads and 1 ml of cold methylene chloride: isopropanol: methanol (25:10:65, v/v/v) + 0.1% butylated hydroxytoluene (BHT). The mixture was incubated at –20°C for 1 h and centrifuged at 13,000 rpm for 10 min 20 µL of the supernatant was diluted 1/10 in extraction solvent before MS analysis. UPLC was performed using a 1.7 μm particle, 2.1 mm × 100, CSH C18 Column (Waters, Milford, MA, United States) coupled to a quadrupole TOF mass spectrometer (AB SCIEX, TripleTOF 5600) operated in information-dependent MS/MS acquisition mode. LC and MS conditions were developed by our group and described previously by Choi et al. (Choi et al., 2015) with some adjustments. For positive ion mode LC-QToF-MS/MS, the mobile phases consisted of (A) 60:40 (v/v) acetonitrile: water with ammonium formate (10 mM) and formic acid (0.1%) and (B) 90:10 (v/v) isopropanol: acetonitrile with ammonium formate (10 mM) and formic acid (0.1% formic acid). For analyses run in the negative ion mode, ammonium acetate (10 mM) was used as the modifier. Quantification of lipid species was performed using MultiQuant Software version 3.0.2 (SCIEX), after annotation in PeakView Software Version 1.2 (SCIEX) based on accurate masses and retention times for each lipid. The library of lipid profiling for identification was introduced by Cajka et al. (Cajka et al., 2017).

### Bile Acid Analysis

Plasma samples collected post-euthanasia (20 μL, *n* = 15–18 per genotype-diet group) were spiked with 0.1 ng of cholic acid-d_4_ internal standard (Cayman Chemical, Ann Harbor, MI, United States) per µL of plasma. 1 ml of ice-cold acetonitrile was added, and the mixture was vortexed and centrifuged at 13,000 rpm for 10 min. The supernatant was evaporated under vacuum and reconstituted in 50% MeOH.

Liver samples without gallbladder (25 mg, *n* = 15–18 per genotype-diet group) were homogenized in 1 ml of solvent (isopropanol/water, 2:1, v/v with 0.1% formic acid) containing 1.6 ng/ml of cholic acid-d_4_ internal standard. Samples were homogenized using a counter-top bullet blender for 5 min and centrifuged at 13,000 rpm for 5 min. The supernatants were filtered with OSTRO phospholipid removal plate (Waters, Milford, MA, United States), evaporated under vacuum and reconstituted in 50% MeOH.

Left and right hippocampus were pooled, ground in liquid nitrogen and freeze-dried. The samples were weighed and spiked with 1 pg of cholic acid-d_4_ internal standard per mg of hippocampus. Approximately 8 mg of hippocampus (dry weight, *n* = 13–17 per genotype-diet group) were homogenized with 1:30 μL (m/v) of 50% MeOH using a counter-top bullet blender for 10 min and centrifuged at 15,000 rpm for 20 min and supernatants used for HPLC analysis.

UPLC was performed using a 1.7 μm particle, 2.1 mm × 100, CSH C18 column (Waters, Milford, MA, United States) coupled to a hybrid triple quadrupole linear ion trap mass spectrometer (AB SCIEX, 4000 QTRAP). LC and MS conditions were developed by our group and described in the Supplemental data. BAs were identified by matching their retention time, isotopic pattern, exact mass of the [M-H]^-^ ion and fragmentation pattern with those of authentic standards (IROA Technologies, Sea Girt, NJ, United States). SRM transitions used for quantification are listed in (Supplementary Table S1) and additional parameters such as collision energy are listed in (Supplementary Table S2).

100% of the mice had hepatic and plasma BA above the detection limit and 82% (52 out of 63 mice) had hippocampal BA above the detection limit, i.e. 74% of the WT mice vs. 89% of the FXR^Liver−/−^ mice.

### XN and Metabolites Concentrations in Liver and Plasma

Liver and plasma extracts (*n* = 15–17 per genotype-diet group) were analyzed for XN and metabolites by LC-MS/MS using a hybrid triple quadrupole linear ion trap mass spectrometer (AB SCIEX, 4000 QTRAP). Analytes were separated by UPLC carried out using a 2.1 × 50 mm Agilent Zorbax 300 SB-C8 3.5 μm column (Agilent, Santa Clara, CA, United States). Each run lasted 6 min at a flow rate of 0.4 ml/min. The elution gradient started at 30% solvent B (0.1% formic acid in acetonitrile) in solvent A (0.1% formic acid in water) and was increased to 60% solvent over the initial 1.5 min. The gradient was held at 60% for 1 min, increased to 100% B for 0.5 min, held at 100% B from 3.0 to 3.8 min, then dropped to 30% B in 0.1 min. The column was equilibrated for 2.1 min until 6.0 min. SRM transitions for quantification were m/z 353 → 119 for XN and isoxanthohumol (IX), m/z 339 → 219 for 8-prenylnaringenin (8 PN), m/z 355 → 249 for α,β-dihydroxanthohumol (DXN), and m/z 341 → 235 for O-desmethyl-α,β-dihydroxanthohumol (DDXN).

### RNA Sequencing and Analysis

RNA was prepared from liver samples (n = 4-5 per genotype-diet group) and sequenced as previously described (Singh et al., 2018). All samples were processed and analyzed in parallel. Sequence quality was assessed by FastQC. Reads were aligned by Hisat2 (Kim et al., 2019) and Samtools (Li et al., 2009). A gene count matrix was generated byStringtie (Pertea et al., 2015). Two data sets, HFD (control) and HFD-XN (treatment) were derived from the gene count matrix. Each set was analyzed in parallel by DESeq2 (Love et al., 2014) for differential expression (DE) calculation. DE was calculated for FXR^Liver−/−^ mutant over wildtype. PCA plots were generated using the DESeq2 package. Benjamini-Hochberg multiple-test correction was applied to control for the number of false positives with an adjusted 5% statistical significance threshold (Benjamini and Hochberg, 1995). A heatmap was created using the pheatmap package in R (version 3.6). Functional annotation clustering was achieved in Network Analyst v3.0 using the Kyoto Encyclopedia of Genes and Genomes (KEGG) database.

### Real-Time PCR

RNA samples from mouse liver (*n* = 4-5 per genotype-diet group) were reverse-transcribed using the High-Capacity cDNA Reverse Transcription Kit (Applied Biosystems, Waltham, MA, United States). universal SYBR^®^ Green Supermix (Bio-Rad, Hercules, CA, United States) was used following the manufacturer’s protocol and amplifications were performed using the ABI Prism 7300 Real-Time PCR System (Applied Biosystems, Waltham, MA, United States). Each sample had two technical replicates. Gene expression was normalized to levels of Polymerase-II. Relative gene expression was calculated using the 2^−ddCt^ method. All primers were purchased from IDT technologies (Coralville, IA, United States) and are listed in (Supplementary Table S3).

### Statistical Analysis

Statistical data were analyzed in SAS version 9.4 (SAS Ins. Inc., Cary, NC). Plasma leptin, AST, and ALT concentrations, intestinal gene expression, and liver receptor data were not normally distributed and could not be normalized through transformation. Therefore, these parameters were analyzed using the non-parametric Wilcoxon rank sum test after checking for interactions. We categorized values into elevated and normal and used Fisher’s exact test to compare treatment groups. BAs and XN concentrations were not normally distributed but rather distributed logarithmically to the base 10, where 1 is equal to 10, two is equal to 100, and 3 is equal to 1000, and were analyzed on that scale. In addition, we categorized BA values into elevated and normal and compared treatment groups using Fisher’s exact test. The remaining lipid data were analyzed without transformation. The effect of XN-treatment was evaluated separately for WT and FXR^Liver−/−^ mice using a generalized linear model in PROC GLM with XN-treatment, sex, and their interaction, because FXR^Liver−/−^ mice had larger variance estimates than WT mice. The effects of genotype and sex were evaluated in untreated mice using a generalized linear model in PROC GLM with genotype, sex, and their interactions, as XN modified the effect of genotype and sex. All statistical tests were two-sided. Significance was declared at *p* ≤ 0.05. Correlations were tested by calculating non-parametric Spearman’s correlation coefficient, r.

## Results

### Sex Influences XN Metabolism in WT and FXR^Liver−/−^ Mice

During the course of the study, weight gain in HFD-fed WT and FXR^Liver−/−^ mice were comparable (Table 1). HFD-fed males gained more body weight than females (Supplementary Figure S2); this effect was significant in WT mice (*p* < 0.0001) but not in FXR^Liver−/−^ mice (*p* = 0.1). To ensure oral bioavailability of XN in WT and FXR^Liver−/−^ mice, we measured liver and plasma concentrations of XN and its metabolites in XN-treated mice. Oral bioavailability of XN was comparable in both genotypes (Supplementary Table S4), but IX, a product of XN isomerization, reached higher concentrations in the liver of WT mice compared to FXR^Liver−/−^ mice. Moreover, we observed sex-related differences as females had significantly higher concentrations of XN and IX than males (Table 2). Since there was no difference in food intake, this observation is likely a result of the lower body weight in females compared to males. α,β-Dihydroxanthohumol (DXN), a bacterial metabolite of XN (Paraiso et al., 2019) was not affected by sex or genotype, while 8-prenylnaringenin (8PN) hepatic concentrations were elevated in male FXR^Liver−/−^ mice. These observations suggest an influence of sex on XN pharmacokinetics, likely due to the differences in weight and volume of distribution between males and females.

**TABLE 1:** A list of metabolic parameters measured in WT and FXR^Liver−/−^ mice upon 10 weeks (^a^) or 12°weeks of HFD ± XN.

	WT	WT XN	FXR^Liver−/−^	FXR^Liver−/−^ XN
Initial body weight (g)	22.28 ± 0.9	22.18 ± 0.71	22.57 ± 0.73	22.32 ± 0.52
Body weight gain^a^ (g)	12.22 ± 1.62	11.71 ± 1.73	16.07 ± 1.63	15.72 ± 1.02
Body weight gain (g)	15.25 ± 1.38	14.62 ± 1.45	16.22 ± 1.54	16.24 ± 1.18
Fasting glucose^a^ (mg/dl)	200.73 ± 13.55	198 ± 9.59	200.5 ± 10.82	203.81 ± 6.2
Liver weight (g)	1.11 ± 0.08	1.09 ± 0.12	1.35^#^ ± 0.09	1.23 ± 0.07
% Liver weight (% body weight)	2.95 ± 0.11	2.84 ± 0.17	3.50^#^ ± 0.18	3.41 ± 0.19
AST (U/mL)	0.32 ± 0.09	0.28 ± 0.08	1.46^#^ ± 0.6	0.24^*^ ±0.08
ALT (U/mL)	0.53 ± 0.14	0.38 ± 0.1	0.83 ± 0.39	0.33 ± 0.08
Food intake (g/day)	3.05 ± 0.34	3.40 ± 0.17	2.51 ± 0.05	2.79 ± 0.06
Leptin (ng/ml)	30.14 ± 6.99	29.75 ± 4.03	66.2 ± 15.36	43.38 ± 10.52

Data displayed as mean ± SEM. Significant differences are marked as ^*^ *p* < 0.05, ^**^ *p* < 0.01, ^***^ *p* < 0.001 for effect of XN treatment, ^#^ *p* < 0.05, ^##^ *p* < 0.01, ^###^ *p* < 0.001 for genotype comparison, ^&^ *p* < 0.05, ^&&^ *p* < 0.01, ^&&&^ *p* < 0.001 for gender comparison.

**TABLE 2:** Concentrations of XN and metabolites (IX, 8PN, DXN) in the plasma and liver of females vs. males HFD-fed WT and FXR^Liver−/−^ mice.

	Plasma (nM)			
	**Female WT XN**	**Male WT XN**	**Female FXR^Liver−/−^ XN**	**Male FXR^Liver−/−^ XN**
XN	22.61 ± 4.4	16.94 ± 3.2	30.45 ± 5.3	14.65^&^ ± 1.8
IX	16.0 ± 4.0	8.73^&^ ± 2.0	13.55 ± 3.2	8.78 ± 1.6
DXN	1.90 ± 0.4	3.02 ± 1.2	1.36 ± 0.45	3.60 ± 1.89
	**Liver (nmol/g)**			
	**Female WT XN**	**Male WT XN**	**Female FXR^Liver−/−^ XN**	**Male FXR^Liver−/−^ XN**
XN	0.28 ± 0.05	0.16^&^ ± 0.03	0.23 ± 0.08	0.29^&^ ± 0.05
IX	1.53 ± 0.35	0.68^&^ ± 0.08	0.58^##^ ± 0.09	0.65 ± 0.05
8PN	0.04 ± 0.01	0.05 ± 0.01	0.06 ± 0.03	0.13^&,&&^ ± 0.04

Data displayed as mean ± SEM (*n* = 7–10 per group). ^#^ *p* < 0.05, ^##^ *p* < 0.01 for genotype comparison, ^&^ *p* < 0.05, ^&&^ *p* < 0.01 for gender comparison.

### XN Ameliorates HFD-Induced Liver Damage

**FIGURE 1:**
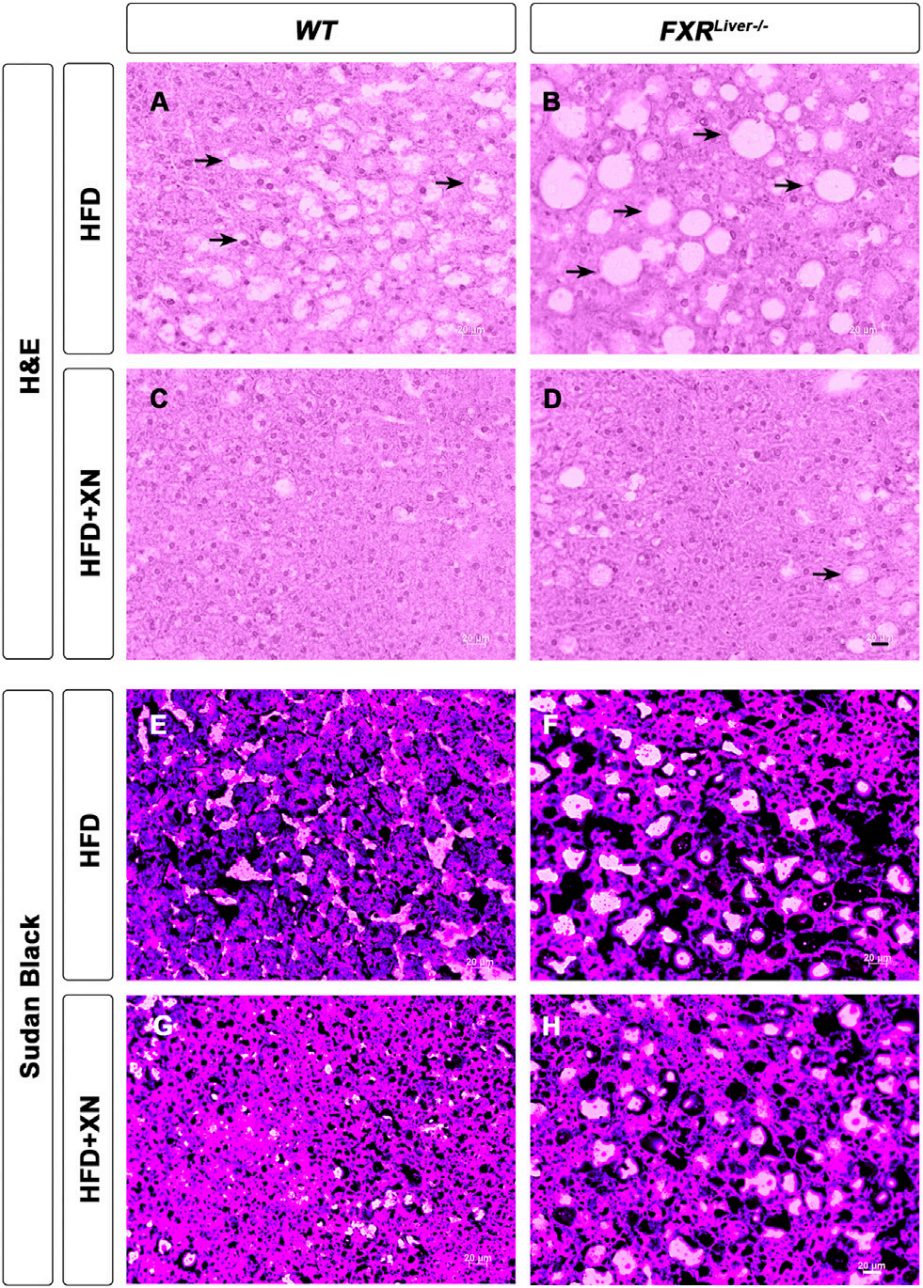
XN prevents HFD-induced hepatic steatosis. Representative liver histology by H&E **(A-D)** and Sudan Black **(E-H)** of male WT and FXR^Liver−/−^ mice fed HFD ± XN. Arrows indicate vacuoles, a characteristic structure of hepatic steatosis.

To assess if the HFD successfully induced NAFLD, we examined liver sections from three representative male mice per treatment group. Hematoxylin and eosin (H&E) stained liver sections showed hepatic steatosis in the form of vacuoles with a clear appearance in HFD-fed WT and FXR^Liver−/−^ mice (Figures 1A,B). We observed a reduction in number and size of these vacuoles in both genotypes in XN-treated mice (Figures 1C,D). Sections stained with Sudan black confirmed an increase in lipid vacuoles in FXR^Liver−/−^ mice compared to WT (Figures 1E,F), which was reversed in XN-treated mice (Figures 1G,H, Supplementary Figure S3). Another marker of NAFLD is the proportion of liver weight (LW) over total body weight (LW%). After 12 weeks on the HFD, untreated FXR^Liver−/−^ mice exhibited increased LW (*p* = 0.02) and LW% (*p* = 0.03) than WT mice (Table 1). In addition, males had increased LW than females in WT mice (*p* = 0.001) and FXR^Liver−/−^ mice (*p* < 0.0001, Supplementary Figure S4A). Fasting glucose was also elevated in males compared to females in both genotypes (Supplementary Figure S4B).

To measure the extent to which the steatosis had resulted in liver tissue damage, we measured aspartate aminotransferase (AST) and alanine aminotransferase (ALT) enzymatic activities in liver homogenates. Absence of hepatic FXR might promote liver tissue damage as AST levels were increased in untreated FXR^Liver−/−^ compared to WT mice (*p* = 0.02, Table 1). XN reduced AST levels in treated FXR^Liver−/−^ mice (*p* = 0.03). Plasma leptin concentrations were elevated in FXR^Liver−/−^ mice but differences in leptin and food intake among groups were not significant. These results suggest that HFD-induced NAFLD is accentuated in absence of hepatic FXR and the severity of the hepatic steatosis is attenuated by XN supplementation.

### XN Ameliorates HFD-Induced Lipid Accumulation

**FIGURE 2:**
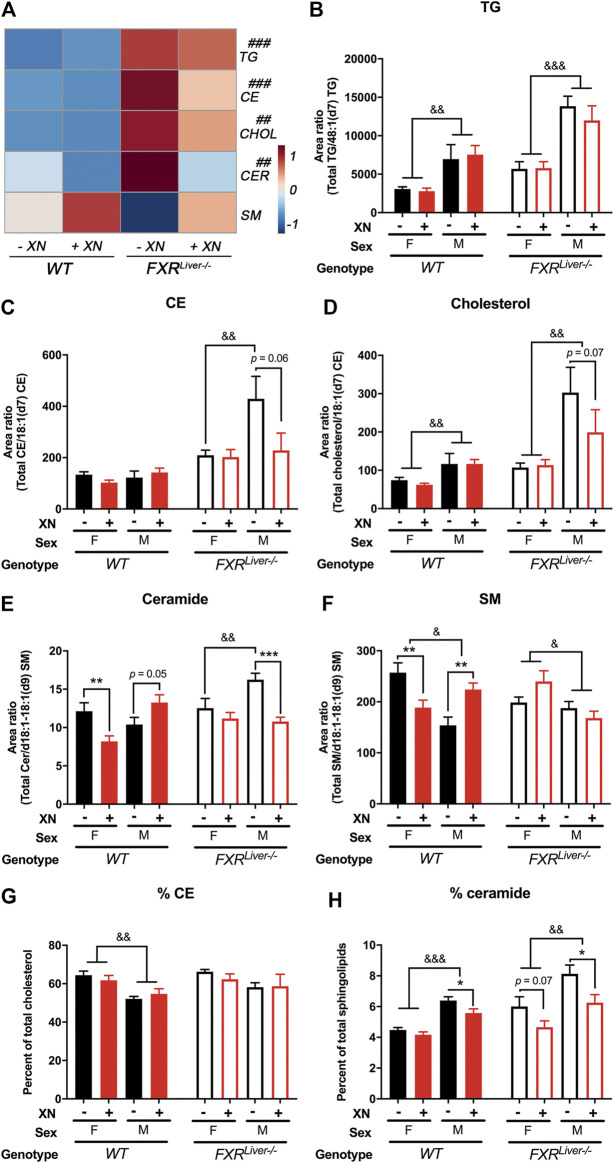
XN prevents HFD-induced ceramide accumulation **(A)** Heatmap of hepatic triglycerides (TG), cholesterol esters (CE), free cholesterol (CHOL), ceramides (CER) and sphingomyelins (SM) in HFD-fed WT and FXR^Liver−/−^ mice untreated or treated with XN. Total relative abundance of **(B)** TG **(C)** CE **(D)** free cholesterol **(E)** ceramide and **(F)** SM in the liver of WT and FXR^Liver−/−^ mice untreated or treated with XN. Proportion of **(G)** CE and **(H)** ceramide in the liver of WT and FXR^Liver−/−^ mice untreated or treated with XN. Values are mean ± SEM (n = 7–10 per group). *p < 0.05, **p < 0.01, ***p < 0.001 for effect of XN treatment; #*p* < 0.05, ##*p* < 0.01, ###*p* < 0.001 for genotype comparison; ^&^ *p* < 0.05, ^&^ *p* < 0.01, ^&^ *p* < 0.001 for gender comparison.

We annotated and measured the relative abundances of 116 individual hepatic lipids including triglycerides (TG), free cholesterol, esterified cholesterol (CE), ceramides and sphingomyelins (SM; Figure 2A). We observed sex, genotype and XN-dependent effects on lipid composition (Figures 2B–H).

Hepatic TG were increased in female FXR^Liver−/−^ compared to their WT counterparts, but male FXR^Liver−/−^ mice displayed the most severe hepatic steatosis. Male FXR^Liver−/−^ mice had elevated hepatic TG, cholesterol, CE and ceramides compared to female FXR^Liver−/−^ mice and compared to male WT mice (Figures 2B–E). The proportion of CE over total cholesterol (%CE) and the proportion of ceramide over total sphingolipids (%ceramide) was higher in FXR^Liver−/−^ males compared to WT males (Figures 2G,H). These data indicate that male mice are more responsive to diet-induced hepatic steatosis in the absence of FXR signaling in the liver.

In FXR^Liver−/−^ mice, XN had a predominant effect in male mice, which exhibited the highest hepatic lipid accumulation. Total CE (*p* = 0.06), cholesterol (*p* = 0.07) and ceramides (*p* = 0.0005) were decreased in XN-treated male FXR^Liver−/−^ mice compared to the untreated mice. The %ceramide, a measure of the proportion of ceramides among other sphingolipids, correlated better with histological improvements than ceramide abundances. XN treatment decreased %ceramide in male WT (*p* = 0.01) and male FXR^Liver−/−^ mice (*p* = 0.02); both groups had the most elevated %ceramide among the untreated groups. SM abundances followed a trend opposite to that of other lipids and were increased in XN-treated WT males (Figure 2F). These data suggest that XN regulates lipid metabolism via pathways independent of hepatic FXR signaling.

### XN Ameliorates HFD-Induced Dysfunctional BA Metabolism

We screened for 34 individual BAs in the plasma, liver and hippocampus of WT and FXR^Liver−/−^ mice using UPLC-MS/MS. Hippocampal BAs were measured to investigate BA retention in tissues deficient in BA detoxification and export mechanisms. Fifteen BAs were detected and quantified in the liver, 12 BAs in the plasma and 7 BAs in the hippocampus.

**FIGURE 3:**
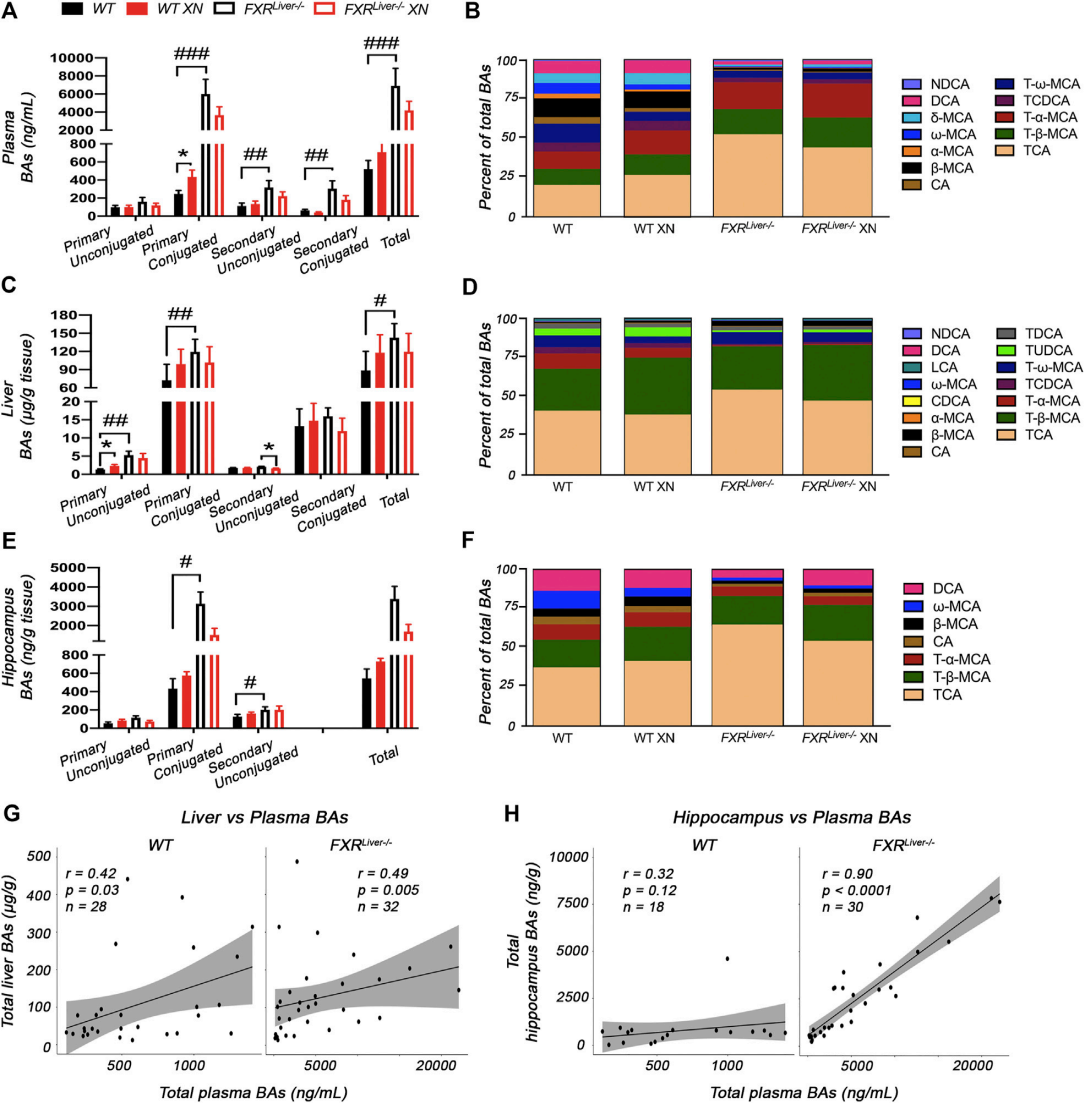
XN modulates BA composition **(A)** Total BAs and **(B)** composition of the BA pool in the plasma of HFD-fed WT and FXR^Liver−/−^ mice **(C)** Total BAs and **(D)** composition of BAs in the liver of HFD-fed WT and FXR^Liver−/−^ mice **(E)** Total BAs and **(F)** composition of BAs in the hippocampus of HFD-fed WT and FXR^Liver−/−^ mice **(G)** Correlations between liver and plasma BAs in WT and FXR^Liver−/−^ mice **(H)** Correlations between hippocampus and plasma BAs in WT and FXR^Liver−/−^ mice. Bar graphs values are mean ± SEM (n = 15–18 per group). *p < 0.05, **p < 0.01, ***p < 0.001 for effect of XN treatment; #*p* < 0.05, ##*p* < 0.01, ###*p* < 0.001 for genotype comparison. Abbreviations: chenodeoxycholic acid (CDCA), cholic acid (CA), deoxycholic acid (DCA), lithocholic acid (LCA), muricholic acid (MCA), nordeoxycholic acid (NDCA), taurochenodeoxycholic acid (TCDCA), taurocholic acid (TCA), tauromuricholic acid (T-MCA), taurodeoxycholic acid (TDCA), tauroursodeoxycholic acid (TUDCA).

FXR^Liver−/−^ had higher BA concentrations in plasma (*p* < 0.0001, Figure 3A) and liver (*p* = 0.01, Figure 3C) than WT mice, while an increase in hippocampal BAs was observed in FXR^Liver−/−^ males only (*p* = 0.04, Supplementary Figure S5). The most severe BA accumulation occurred in the plasma of FXR^Liver−/−^ mice, with a 7-fold increase in total BAs vs. 2-fold increase in the liver. The change was driven by an increase in primary conjugated BAs (Figures 3A–D). Hippocampal BA retention was more pronounced than hepatic BA retention as FXR^Liver−/−^ mice exhibited 9-fold increase in total hippocampal BAs compared to WT (Figures 3E,F) indicating passage of BAs through a possibly altered blood brain barrier (BBB). These data suggest that hepatic mechanisms of BA efflux remained more efficient than cerebral mechanisms of BA efflux in FXR^Liver−/−^ mice. This is further supported by our observation that, in FXR^Liver−/−^ mice, plasma BAs were more strongly correlated to hippocampal BAs (r = 0.90, *p* < 0.0001) than to hepatic BAs (r = 0.49, *p* = 0.005, Figures 3G,H). BA pool composition was also modified in FXR^Liver−/−^ mice. The percentage of plasma primary conjugated BAs over total BAs was 50% in WT mice vs. 85% in FXR^Liver−/−^ mice (Figure 3B). Hepatic conjugation of BAs improves their hydrophilicity and reduces their toxicity suggesting that FXR^Liver−/−^ mice developed a metabolic mechanism to counter BA-mediated toxicity.

**FIGURE 4:**
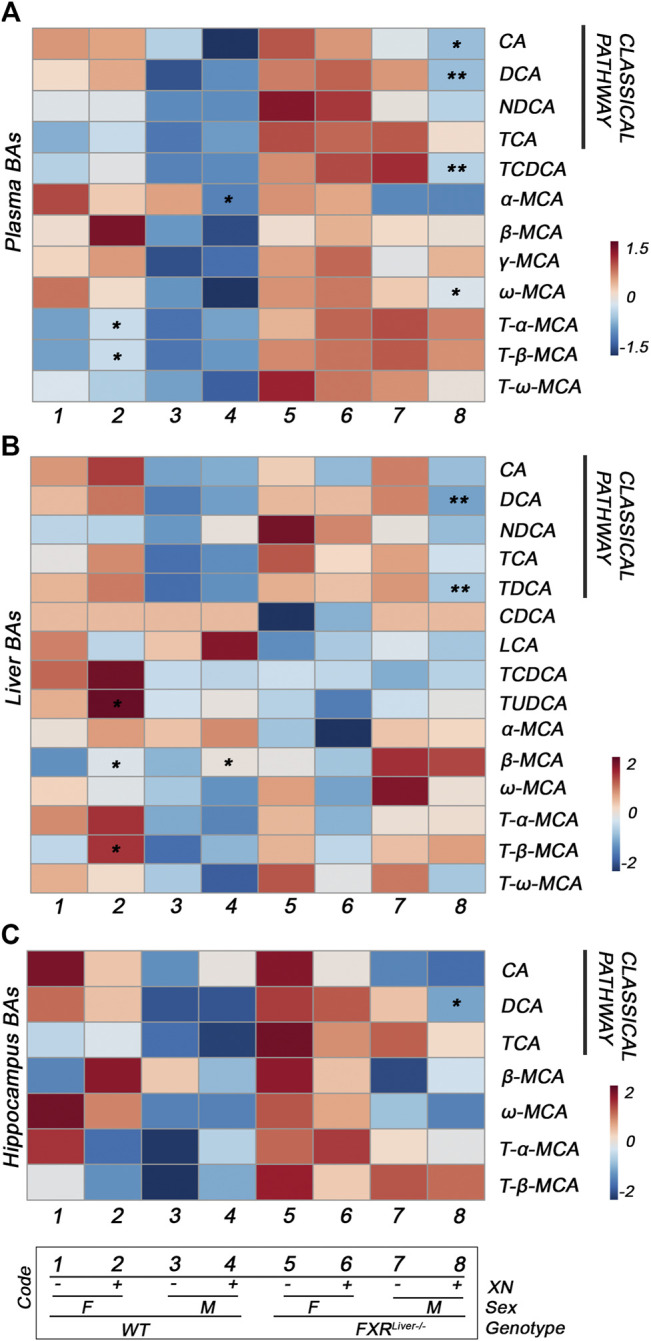
XN differentially modulates classical and alternative pathways of synthesis in WT vs. FXR^Liver−/−^ mice. Heatmaps of individual BA concentrations in the plasma **(A)**, liver **(B)** and hippocampus **(C)** of HFD-fed WT and FXR^Liver−/−^ mice. *p < 0.05, **p < 0.01, ***p < 0.001 for effect of XN treatment (*n* = 7–10 per group). Abbreviations: chenodeoxycholic acid (CDCA), cholic acid (CA), deoxycholic acid (DCA), lithocholic acid (LCA), muricholic acid (MCA), nordeoxycholic acid (NDCA), taurochenodeoxycholic acid (TCDCA), taurocholic acid (TCA), tauromuricholic acid (T-MCA), taurodeoxycholic acid (TDCA), tauroursodeoxycholic acid (TUDCA).

XN treatment promoted BA synthesis in WT mice but attenuated BA accumulation in FXR^Liver−/−^ mice. XN effect on BA concentrations was independent of sex in WT mice. XN supplementation of WT mice resulted in increased plasma primary conjugated BAs (*p* = 0.03, Figure 3A) and increased hepatic primary unconjugated BAs (*p* = 0.02, Figure 3C). These observations are in accordance with previous reports that XN induces CYP7A1 and hepatic BA synthesis in WT mice resulting in increased BA concentrations (Paraiso et al., 2020). DCA, TCA, β-MCA and FXR antagonists, T-α-MCA and T-β-MCA, were increased in the liver and/or plasma of XN-treated WT mice (Figures 4A,B). By contrast, XN treatment resulted in decreased BA concentrations in FXR^Liver−/−^ mice. CA and CA-derived BAs including DCA, TCA and TDCA were decreased in the liver of FXR^Liver−/−^ mice in both sexes, with males exhibiting more significant changes (Figures 4A–C). CA, DCA and ω-MCA were decreased in the plasma of XN-treated male FXR^Liver−/−^ mice.

In summary, XN reduced most individual BAs of the classical pathway in FXR^Liver−/−^ mice and increased BAs from the alternative pathway of synthesis in WT mice (Figures 4A,B; Supplementary Figure S6). Since BAs did not reach pathological concentrations in WT mice, these results suggest an adaptation of XN mechanism of action to the pathophysiological conditions and the possible activation of BA receptors independent of FXR. These observations further support a genotype-specific differential modulation of metabolism by XN.

### XN Ameliorates HFD-Induced Hepatic Gene Profiles

**FIGURE 5:**
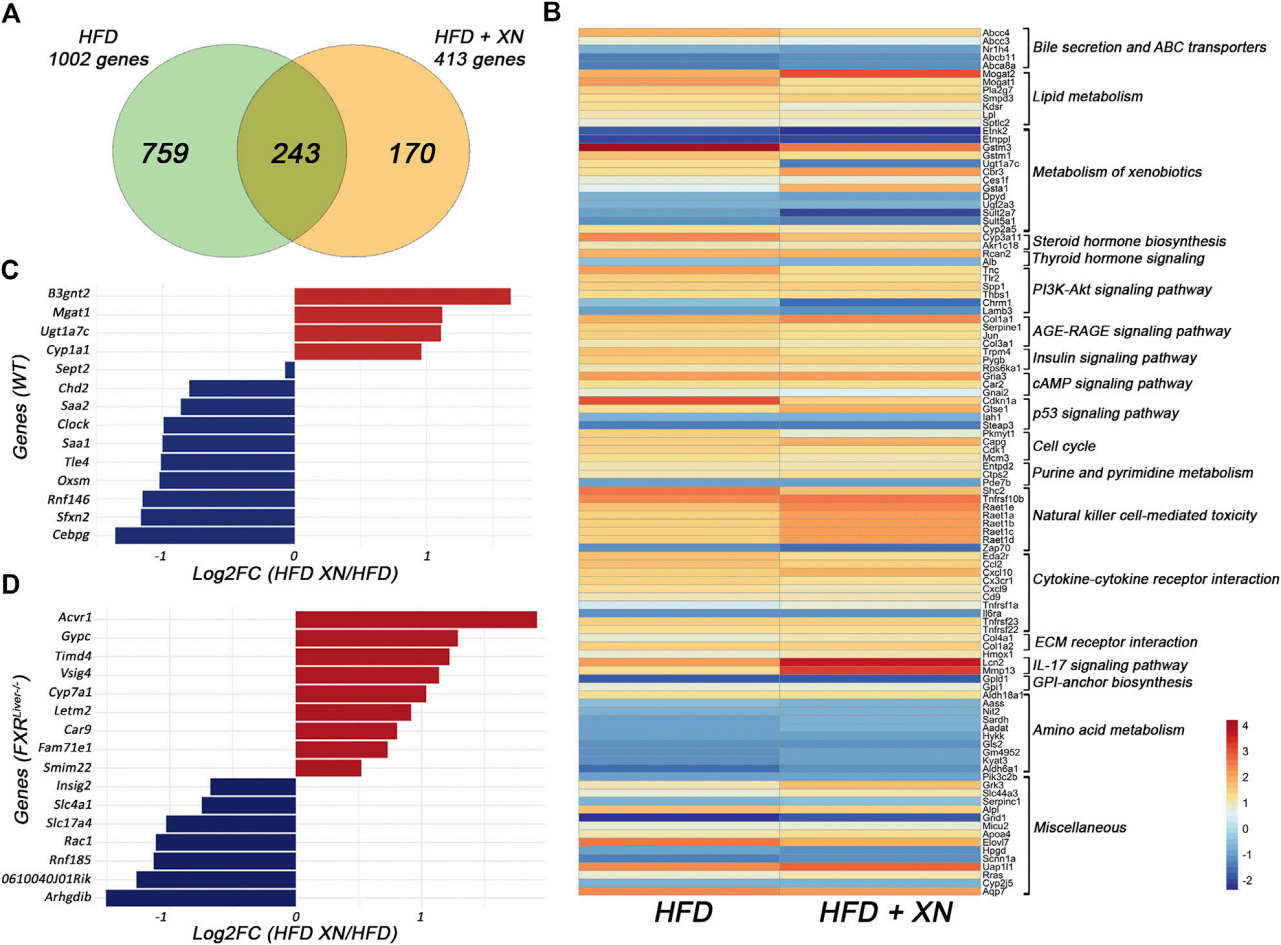
XN alters hepatic gene profiles **(A)** Venn diagram comparing genes in HFD-fed mice vs. HFD-fed mice treated with XN **(B)** Relative expression of 106 shared genes classified according to KEGG pathway (Log2FC of FXR^Liver−/−^ ± XN/WT) **(C)** Genes regulated by XN in WT mice **(D)** Genes regulated by XN in FXR^Liver−/−^ mice. Values are mean of n = 4-5 mice (males and females) per group.

We analyzed changes in global gene expression profiles in hepatic tissue of HFD-fed WT and FXR^Liver−/−^ mice. Since a smaller subset of samples was sequenced, male and female mice RNA sequencing data were pooled to increase the power of the analysis. Functional annotation clustering revealed that genes differentially affected by HFD in WT and FXR^Liver−/−^ mice can be classified into two main functional groups: genes involved in metabolic processes vs. genes involved in inflammation and carcinogenic processes (Supplementary Table S5). The comparison between XN-treated and untreated FXR^Liver−/−^ mice revealed 243 shared genes, with 759 features unique to untreated mice and 170 features unique to XN-treated mice (Figure 5A). Within the shared features, XN supplementation impacted several gene networks including lipid metabolism (Mgat2, Sptlc2, Smpd3), ABC transporters involved in BA transport (Abcc4, Abcc3, Abcb11), metabolism of xenobiotics with genes involved in phase I and II metabolism (Gsta1, Gstm3, Ugt1a7c, Sult2a7), PI3K-Akt signaling pathway (Tnc, Tlr2, Spp1, Thbs1, Lamb3), cytokine-cytokine receptor interactions (Ccl2, Cxcl9/10, Cd9, Tnfrsf1a) and amino acid metabolism (Sardh, Aadat, Kyat3) (Figure 5B).

In WT mice, presence of XN was associated with increased expression of genes involved in lipid and xenobiotic metabolism such as Mgat1, Cyp1a1, Ugt1a7c, and decreased expression of genes involved in energy metabolism (Clock, Rnf146) and inflammation (Cebpg, Saa1, Saa2) (Figure 5C; Table 3). CCAAT-enhancer binding proteins (C/EBP) interact with the proximal promoter of the Saa genes and regulate hepatic expression of SAA (Ray et al., 1995). The concurrent decrease in Cebpg and Saa expression suggests that XN-mediated repression of Saa1 and Saa2 is mediated by inhibition of Cebpg expression.

**TABLE 3:** Hepatic genes regulated by XN in WT and FXR^Liver−/−^ mice and their roles in metabolic function.

Genes	Log2FC	Definition	Function	Ref
**WT mice (HFD + XN/HFD)**				
B3gnt2	+ 1.64	Beta-1,3-N-acetylglucosaminyltransferase	Glycosphingolipid biosynthesis	Togayachi et al. (2010)
Mgat1	+ 1.12	Monoacylglycerol acyltransferase	Triglyceride synthesis	Lee and Kim. (2017)
Ugt1a7c	+ 1.1	Uridine 5′-diphospho-glucuronosyltransferase 1A7c	Lipid and xenobiotic metabolism	Guillemette. (2003)
Cyp1a1	+ 0.96	Cytochrome P450 1A1	Lipid and xenobiotic metabolism	Stejskalova and Pavek. (2011)
Sept2	–0.07	Septin 2	Apoptosis and cell proliferation	Cao et al. (2015)
Chd2	–0.79	Chromodomain-helicase-DNA-binding protein 2	Epigenetic signature during liver development	Lu et al. (2012)
Clock	–0.99	Circadian locomotor output cycles kaput protein	Energy metabolism and obesity	Turek et al. (2005). Vieira et al. (2014)
Saa1	–1.00	Serum amyloid a protein 1	Inflammation and systemic complications of obesity	Poitou et al. (2005). Jumeau et al. (2019)
Saa2	–0.86	Serum amyloid a protein 2		
Tle4	–1.01	Transducin-like enhancer protein 4	Transcriptional corepressor associated with type 2 diabetes	Ali, (2013)
Oxsm	–1.02	3-Oxoacyl-ACP synthase II	Fatty acid metabolism	Gao et al. (2019)
Rnf146	–1.15	E3 ubiquitin-protein ligase Rnf146	Energy metabolism	Matsumoto et al. (2017)
Sfxn2	–1.16	Sideroflexin2	Mitochondrial biogenesis	Mon et al. (2019)
Cebpg	–1.36	CCAAT/enhancer binding protein gamma	Transcriptional regulation of adipogenesis and inflammation	Ray et al. (1995); Tanaka et al. (1997)
**FXR^Liver−/−^ mice (HFD + XN/HFD)**				
Acvr1	+ 1.92	Activin a receptor type 1	TGF-β signaling pathway	Rao et al. (2017)
Gypc	+ 1.29	Glycophorin C	Membrane properties of erythrocytes	Yiangou et al. (2016)
Timd4	+ 1.22	T-cell immunoglobulin and mucin domain containing 4	Adaptative immunity	Albacker et al. (2010). Dai et al. (2020)
Vsig4	+ 1.14	V-set and immunoglobulin domain-containing 4	Macrophage-mediated hepatic inflammation	Li et al. (2019)
Cyp7a1	+ 1.04	Cholesterol 7 alpha-monooxygenase	Cholesterol and bile acid metabolism	Chiang, (2009)
Letm2	+ 0.92	Leucine zipper and EF-hand containing transmembrane 2	Mitochondrial ion uptake	Waldeck-Weiermair et al. (2011)
Car9	+ 0.8	Carbonic anhydrase 9	Hypoxia-inducible	Olive et al. (2001)
Fam71e1	+ 0.73	Family with sequence similarity 71 member E1		
Smim22	+ 0.52	Small integral membrane protein 22	Cell proliferation	Polycarpou-Schwarz et al. (2018); Li et al. (2019)
Insig2	–0.68	Insulin induced gene 2	Lipid and glucose metabolism	Dong and Tang, (2010)
Slc4a1	–0.74	Solute carrier family 4A1	Efflux transport	Hediger et al. (2004)
Slc17a4	–1.03	Solute carrier family 17A4		
Rac1	–1.11	Ras-related C3 botulinum toxin substrate 1	Cell proliferation	Choi et al. (2006)
Rnf185	–1.13	E3 ubiquitin-protein ligase Rnf185	Autophagy	Tang et al. (2011)
0610040J01Rik	–1.26	RIKEN cDNA 0610040J01 gene		
Arhgdib	–1.51	Anti-rho guanosine diphosphate dissociation inhibitor beta	Liver fibrosis	Utsunomiya et al. (2007)

In FXR^Liver−/−^ mice, XN induced Vsig4, which attenuates macrophage-mediated hepatic inflammation (Li et al., 2019), Acvr1, which is involved in activin signaling (Rao et al., 2017), and Timd4, that controls adaptive immunity by clearing antigen-specific T-cells (Albacker et al., 2010) (Figure 5D; Table 3). While XN induced increased expression of Cyp7a1, which is involved in the classical pathway of BA synthesis, there were no changes in the expression of genes involved in the alternative pathway of BA synthesis. RhoGDI2, encoded by Arhgdib, is involved in the molecular pathogenesis of liver fibrosis (Utsunomiya et al., 2007) and acts as a positive regulator of Rac1 (Kardol-Hoefnagel et al., 2020). The decreased expression of both genes indicated that XN inhibition of Rac1 transcription might be mediated by repression of Arhgdib in FXR^Liver−/−^ mice (Table 3). These data show that XN promotes lipid and BA metabolism and decreases acute inflammation in WT mice, while XN attenuates inflammation by controlling immune response, inhibits cell proliferation and liver fibrosis in FXR^Liver−/−^ mice.

### XN Induces Gene Expression of FXR-Independent NRs

**FIGURE 6:**
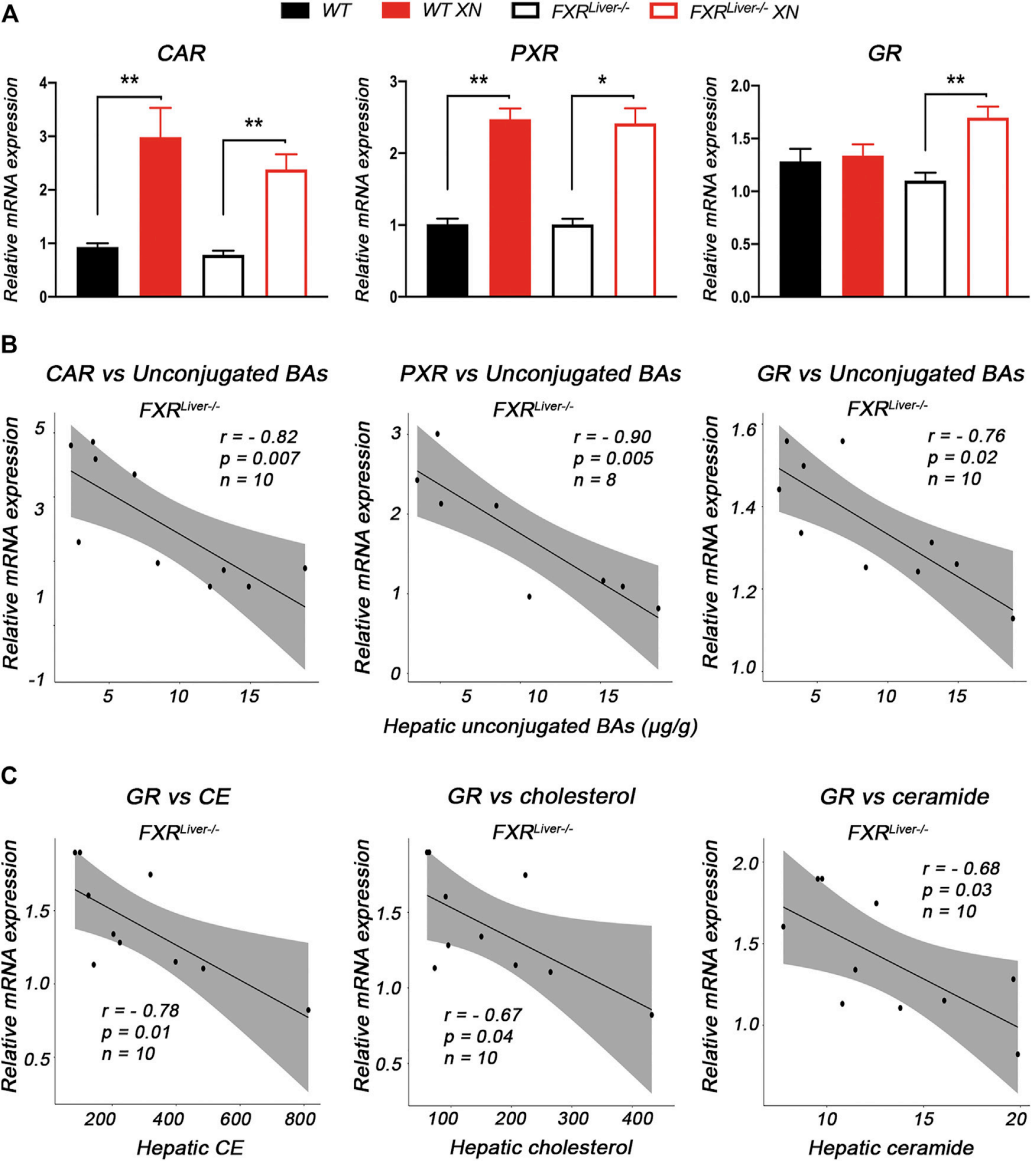
XN induces hepatic expression of FXR-independent NRs **(A)** Quantitative relative expression of CAR, PXR and GR in the liver of HFD-fed WT and FXR^Liver−/−^ mice **(B)** Correlations between relative mRNA expression of CAR, PXR and GR vs. hepatic unconjugated BAs concentrations in FXR^Liver−/−^ mice **(C)** Correlations between relative mRNA expression of GR vs. relative abundances of hepatic CE, cholesterol and ceramide in FXR^Liver−/−^ mice. Bar graphs values are mean ± SEM, *n* = 4-5 mice (males and females) per group. *p < 0.05, **p < 0.01, ***p < 0.001 for effect of XN treatment.

RNA sequencing revealed several genes involved in phase II reactions such as glucuronidation (UGTs), sulfation (SULTs) and glutathione conjugation (GSTs) were regulated by XN. Therefore, we performed a quantitative analysis of the hepatic expression of a panel of NRs known to regulate phase II BA metabolism including CAR, PXR and GR. BAs interact with CAR (Moore et al., 2002), PXR (Staudinger et al., 2001), as well as GR (Tanaka and Makino, 1992), which also regulates biosynthesis and transport of bile salts (Xiao et al., 2016). XN treatment resulted in higher CAR expression in all sex and genotype groups (Figure 6A), although XN effect was stronger in males. XN also induced gene expression of PXR in both WT and FXR^Liver−/−^ mice, while an increase in GR transcript levels was observed in XN-treated FXR^Liver−/−^ mice only (*p* = 0.009, Figure 6A).

Changes in these NR expression profiles were linked to hepatic BA concentrations. Correlation analyses revealed that relative expression levels of these receptors were negatively correlated with unconjugated BAs in the liver absence of FXR (Figure 6B). Additionally, relative expression of GR was negatively correlated with relative abundances of lipids regulated by XN, i.e. CE (r = -0.78, *p* = 0.01), cholesterol (r = -0.67, *p* = 0.04) and ceramide (r = -0.68, *p* = 0.03) in absence of FXR. Collectively, these data suggest that, in absence of hepatic FXR, induction of CAR, PXR and GR is involved in XN-mediated decrease of lipid and BA concentrations.

## Discussion

NRs regulate ligand-activated transcriptional activation of a myriad of genes for the elimination and detoxification of potentially toxic biliary constituents accumulating in cholestasis (Halilbasic et al., 2013). FXR controls the transcriptional activation of several genes involved in the regulation of glucose and lipid metabolism and maintenance of BA homeostasis, thereby protecting the host against liver damage associated with lipid and BA accumulation. BAs act as signaling molecules through BA receptors such as FXR, TGR5, PXR and VDR to regulate TG, cholesterol, glucose, and energy homeostasis (Schaap et al., 2014; Fiorucci and Distrutti, 2015). BAs inhibit their own synthesis mainly via FXR-mediated negative feedback of CYP7A1, the rate-limiting enzyme in the catabolism of cholesterol into BAs (Goodwin et al., 2000). As a result, FXR knockout mice exhibit dyslipidemia (Sinal et al., 2000; Kok et al., 2003) and hepatic steatosis that progresses to NASH (Armstrong and Guo, 2017).

Liver histology revealed lipid vacuoles characteristic of fatty liver disease in FXR^Liver−/−^ mice and elevated hepatic levels of triglycerides, free cholesterol, cholesterol esters and ceramides. The increased liver enzymes indicate liver injury associated with inflammation and NASH. HFD-induced dyslipidemia was aggravated by FXR deficiency with sex differences. Risk factors associated with HFD-induced obesity including fasting glucose and dyslipidemia were more pronounced in males than females. This is in accordance with previous report that male western diet-fed FXR^−/−^ mice had higher hepatic and serum lipids than their female counterparts (Sheng et al., 2017). In fact, NRs play a crucial role in the calibration of sex-specific metabolic pathways and androsterone (Wang et al., 2006) as well as estrogen (Song et al., 2014) were reported to modulate FXR activity. This suggests that interactions between the receptor and gonadal hormones warrant further investigation. The severity of the FXR^Liver−/−^ phenotype was further aggravated by the accumulation of BAs, which regulate several signaling pathways independent from FXR. In our study, elevated BA concentrations in the plasma of FXR^Liver−/−^ mice were accompanied with higher concentrations of BAs in the hippocampus indicating passage of BAs through the BBB. The concentrations measured in HFD-fed FXR^Liver−/−^ mice are comparable to BA concentrations in the blood and brain of FXR^−/−^ mice with hepatic enceph-alopathy (Huang et al., 2015). In fact, in pathological conditions such as acute liver failure and cholestasis, elevated plasma BAs were reported to increase permeability of the BBB (Quinn et al., 2014; McMillin et al., 2016), warranting the investigation of therapeutic alternatives regulating BA concentrations.

XN anti-hyperlipidemic effect was more accentuated in male FXR^Liver−/−^ mice that developed severe dyslipidemia. Our observation that XN and metabolites reached higher concentrations in female mice suggests that pharmacodynamic effect of XN *in vivo* depends more on the severity of the phenotype and less on XN concentrations in biological tissues. Ceramide abundances were heavily influenced by their precursor, SM. The lipotoxicity of ceramides is well-documented (Summers, 2006; Chaurasia and Summers, 2015), but the role of SM in NAFLD and NASH is controversial. Several studies report lower SM levels in NASH patients (Kartsoli et al., 2020). In fact, SM are important components of biological cell membranes (Slotte and Ramstedt, 2007) with no demonstrated intrinsic lipotoxicity. The increase in hepatic SM in XN-treated WT males was accompanied by an increase in hepatic ceramides that did not correlate with metabolic improvements. Therefore, we used the ratio of ceramides over total sphingolipids to estimate ceramide relative abundances more precisely. This ratio was decreased in XN-treated WT and FXR^Liver−/−^ mice.

**FIGURE 7:**
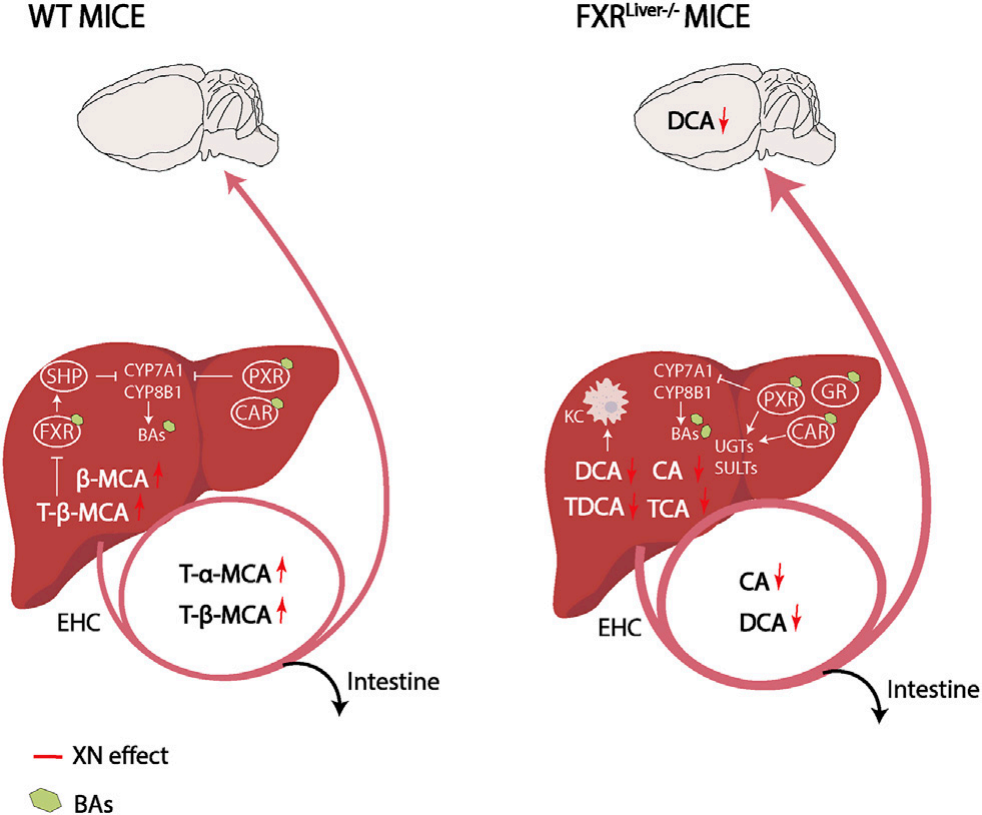
Working hypothesis on XN mechanism of control of BA synthesis and detoxification in WT and FXR^Liver−/−^ mice. XN promotes BA synthesis in WT mice and increases FXR antagonists in the liver and plasma. This results in downstream expression of enzymes involved in BA synthesis, CYP7A1 and CYP8B1. Negative feedback on BA synthesis is exerted by BAs agonists of FXR, PXR and CAR. Compared to WT mice, FXR^Liver−/−^ mice had increased BA synthesis, bigger BA pool sizes and increased passage of BAs through the BBB. In FXR^Liver−/−^ mice, BAs such as CA and DCA impair hepatic Kupffer cells (KCs) activity, which XN might attenuate by decreasing CA and DCA concentrations. XN-mediated activation of PXR and CAR, and GR slows down *de novo* BA synthesis by inhibition of CYP7A1 and induces metabolizing enzymes to stimulate BA excretion. Abbreviations: EHC (Enterohepatic circulation), KC (Kupffer cell).

Taken together, XN protected FXR^Liver−/−^ mice from liver damage, as evaluated by liver transaminase activity, liver histopathology and hepatic expression levels of anti-inflammatory genes. These observations indicate that XN effect is not exclusively mediated by hepatic FXR. We hypothesized that the observed XN effect might also derive from its potential as SBARM and from XN-dependent modulation of BA composition because BAs regulate energy expenditure in mice (Chiang, 2002). In WT mice, XN treatment led to an FXR-dependent increase in the most hydrophilic BAs among which are T-α-MCA and T-β-MCA. FXR antagonists, including T-α-MCA and T-β-MCA, have been reported to improve HFD-induced metabolic dysfunction by inducing thermogenesis and repressing intestinal FXR-FGF15 signaling (Sayin et al., 2013; Jiang et al., 2015). Intestinal FXR-FGF15 signaling regulates CYP7A1 gene expression (Kim et al., 2007). In turn, CYP7A1 regulates T-α-MCA and T-β-MCA synthesis, while CYP8B1 is required for TCA synthesis (Jiang et al., 2015; Chiang, 2009; Qi et al., 2015). In accordance with our results, previous studies have demonstrated that CYP7A1 mRNA levels are induced after XN treatment (Nozawa, 2005; Paraiso et al., 2020). This resulted in the increase of T-α-MCA and T-β-MCA, which repress intestinal FXR-FGF15 signaling to increase hepatic BA synthesis and prevent HFD-induced insulin resistance and obesity (Li et al., 2013; Sayin et al., 2013; Jiang et al., 2015; Gonzalez et al., 2016). Contrary to WT mice, XN decreased hydrophobic BAs such as DCA, CA and their taurine conjugates in FXR^Liver−/−^ mice. Moreover, XN promoted genes that attenuate macrophage-mediated inflammation suggesting a shift toward detoxification in absence of hepatic FXR (Figure 7). Increased concentrations of hydrophobic BAs impairs phagocytosis activity of tissue-resident macrophages called Kupffer cells (KCs), induce neutrophil-mediated inflammation and alter hepatic T-cell immunity (Zhu et al., 2016). The selective depletion of liver-resident KCs restores hepatic insulin sensitivity and improves whole-body and hepatic fat accumulation (Neyrinck et al., 2009; Huang et al., 2010).

The major mechanisms underlying XN-mediated attenuation of liver damage in FXR^Liver−/−^ mice are the reduction of BA concentrations and the mitigation of hepatic inflammation due the activation of NRs CAR/PXR/GR. FXR, PXR and CAR have complementary roles in the protection against BA toxicity (Guo et al., 2003). RNA sequencing revealed that several genes involved in metabolism of xenobiotics such as CYPs, UGTs, SULTs and GSTs were regulated by XN in FXR^Liver−/−^ mice. Conjugation of hydrophilic groups by UGTs, SULTs, and GSTs increases the water solubility of BAs and xenobiotics to facilitate their renal elimination (Garcia et al., 2018). Hepatic xenobiotic-sensing receptors CAR and PXR mediate phase I and II BA metabolism by regulating CYP450s, UGTs, SULTs and GSTs that catalyze synthesis, oxidation, sulfonation and glucuronidation of BAs (Keppler, 2011; Garcia et al., 2018; Lv and Huang, 2020). Phase III clearance of BA is also regulated by FXR, PXR and CAR. Once BAs are transformed into more hydrophilic metabolites in the liver, they are pumped into the bile via efflux transporters BSEP and MRP2 as a route for fecal elimination (Wagner et al., 2005; Garcia et al., 2018). Moreover, GR enhances CAR/PXR-mediated transcriptional regulation of target genes such as UGT1A1 (Sugatani et al., 2005). CAR, PXR and GR are activated by unconjugated BAs such as LCA, CA, DCA and UDCA (Tanaka and Makino, 1992; Staudinger et al., 2001; Moore et al., 2002; Carazo et al., 2017). The strong correlations between these NRs and hepatic unconjugated BA concentrations suggest that BA metabolism in XN-treated FXR^Liver−/−^ mice was primarily regulated by the activation of CAR, PXR and GR. Since the expression levels of these receptors were exclusively correlated with unconjugated BAs, it is conceivable that endogenous BAs might also play a role in the activation of these NRs *in vivo*. Consistent with our observations in FXR^Liver−/−^ mice, taurine conjugated BA species such as TCA are increased in the liver of NASH patients (Lake et al., 2013). The decreased concentrations of hepatic TCA by XN also indicated a normalization of BA metabolism independent from FXR. Due to the affinity of TCA to FXR, it has been hypothesized that TCA is elevated as a compensatory effect to activate the receptor and normalize metabolism (Sheng et al., 2017). GR activation by XN in FXR^Liver−/−^ mice is involved in the lipid-lowering effect of the flavonoid. Partial agonism of GR reverses NAFLD by preventing hepatic TG and cholesterol accumulation (Koorneef et al., 2018). Moreover, the anti-inflammatory effects of GR activation have a positive impact on hepatic lipid accumulation (Rando and Wahli, 2011; Scheschowitsch et al., 2017).

Our results support the hypothesis of a compensatory interaction between FXR, CAR and PXR. XN-mediated induction of CAR and PXR was not FXR-dependent. In the absence of FXR, the complementary regulation by CAR, PXR and GR might be involved in XN-mediated decrease of BAs concentrations. Chronic BA overload and prolonged activation of detoxification pathways may lead to the desensitization of BA-sensing receptors, which would contribute to the chronicity of BA-mediated damage. By improving the efficiency of phase II metabolism and phase III hepatic clearance of BAs, XN alleviated the sustained activation of detoxification pathways, improved BA signaling, lipid metabolism and relieved inflammation. Future studies assessing the pharmacodynamic activity of XN metabolites and quantifying glucuronidated and sulfated BA metabolites are necessary to evaluate XN effect more accurately.

Functional annotation clustering revealed that hepatic genes involved in inflammation and neoplastic processes were altered in FXR^Liver−/−^ mice. Hydrophobic BAs are well-known for their cancer-promoting effects and promote carcinogenesis in several tumor models, including hepatocellular carcinoma, colon cancer, and breast cancer (Nagengast et al., 1995; Debruyne et al., 2001; Kim et al., 2006). Our research sheds new light on the chemopreventive potential of XN and highlights the potential of XN as adjuvant therapy in cancers associated with accumulation of BAs such as bile duct cancer and hepatocellular carcinoma.

## Conclusion

BA synthesis and transport are tightly regulated by BA and xenobiotic-sensing NRs, which regulate genes in synthesis, metabolism and clearance of BAs and play a critical role in BA detoxification. Our current study extends previous research and shows the novel findings that 1) XN ameliorates HFD-induced inflammation and tissue damage in FXR^Liver−/−^ mice; 2) XN improves HFD-induced dysfunctional lipid and BA metabolism via FXR-dependent and independent signaling, including the induction of CAR, PXR and GR. To the best of our knowledge, the potential of XN as adjuvant therapy in the management of cholestatic diseases has not been reported and merits further investigation.

## Data Availability

The datasets presented in this study can be found in online repositories. The names of the repository/repositories and accession number(s) can be found below: https://www.ncbi.nlm.nih.gov/bioproject/PRJNA687670.
