# Supplementary material for: Xanthohumol ameliorates Diet-Induced Liver Dysfunction via Farnesoid X Receptor-Dependent and Independent Signaling
Source: Front Pharmacol. 2021 Apr 20;12:643857. doi: 10.3389/fphar.2021.643857 (PMC8093804; doi:10.3389/fphar.2021.643857)
Supplement: Supplementary file 1 [file datasheet1.docx]

**Supplemental data**

**Xanthohumol ameliorates diet-induced liver dysfunction via farnesoid X receptor-dependent and independent signaling**

Ines L. Paraiso^a,b^, Thai Q. Tran^b^, Armando Alcazar Magana^a,b,c^, Payel Kundu^d^, Jaewoo Choi^a^, Claudia S. Maier^c^, Gerd Bobe^a,e^, Jacob Raber^b,d,f^, Chrissa Kioussi^b^, Jan F. Stevens^a,b^

^a^Linus Pauling Institute, Oregon State University, Corvallis, OR, 97331, USA
^b^Department of Pharmaceutical Sciences, Oregon State University, Corvallis, OR, 97331, USA ^c^Department of Chemistry, Oregon State University, Corvallis, OR, 97331, USA

^d^Department of Behavioral Neuroscience, Oregon Health & Science University, Portland, OR, 97239, USA

^e^Department of Animal & Rangeland Sciences, Oregon State University, Corvallis, OR, 97331, USA

^f^Department of Neurology, Psychiatry and Radiation Medicine, Division of Neuroscience, Oregon National Primate Research Center, Oregon Health & Science University, Portland, OR, 97239, USA

Correspondence and requests for materials should be addressed to C.K (email: Chrissa.kioussi@oregonstate.edu) and J.F.S. (email: [fred.stevens@oregonstate.edu](mailto:fred.stevens@oregonstate.edu))

**Disclaimer:** RNA sequencing data was submitted to NCBI database. BioProject ID: PRJNA687670.

**HPLC–MS/MS bile acid method**

**Materials**

The following bile acids were purchased from Sigma-Aldrich (Saint Louis, MO, USA): cholic acid (CA), chenodeoxycholic acid (CDCA), deoxycholic acid (DCA), glycocholic acid (GCA), glycochenodeoxycholic acid (GCDCA), glycodeoxycholic acid (GDCA), glycoursodeoxycholic acid (GUDCA), hyodeoxycholic acid (HDCA), lithocholic acid (LCA), tauro-β-muricholic acid (T-β-MCA), taurocholic acid (TCA), taurochenodeoxycholic acid (TCDCA), taurodeoxycholic acid (TDCA), taurolithocholic acid (TLCA), tauroursodeoxycholic acid (TUDCA), tauro-ω-muricholic acid (T-ω-MCA), ursodeoxycholic acid (UDCA).

The following bile acids were purchased from IROA Technologies (Sea Girt, NJ, USA): 12-ketochenodeoxycholic acid (12-KCDCA), 7-ketochenodeoxycholic acid (7-KCDCA), 7-ketodeoxycholic acid (7-KDCA), coprocholic Acid/Trihydroxycholestanoic acid (THCA), dehydrocholic acid (DHCA), dehydrolithocholic acid (DHLCA), glycohyodeoxycholic acid (GHDCA), glycolithocholic acid (GLCA), muricholic acid (MCA), nordeoxycholic acid (NDCA), taurocholic acid (TCA), tauro-α-muricholic acid (T-α-MCA), ursocholic acid (UCA), α-muricholic acid (α-MCA), β-muricholic acid (β-MCA), γ-muricholic acid (γ-MCA).

**LC-MS/MS analysis of bile acids**

Bile acids were measured by LC-MS/MS analysis using multiple-reaction monitoring (MRM). Liquid chromatographic separation and mass spectrometric detection were performed using a Shimadzu 20AD system (Shimadzu, Columbia, MD) coupled via electrospray ionization (ESI) interface to a QTRAP 4000 (AB SCIEX, Framingham, MA). The chromatographic separation was performed on an ACQUITY UPLC CSH C18 column (130 Å, 1.7 µm, 2.1 mm X 100 mm) from Waters (Waters Corp, Inc., Milford, MA) at 65 °C and the total flow rate 0.25 mL/min. A gradient with two mobile phases (A, 0.1% formic acid in water; B, acetonitrile with formic acid 0.1% v/v) was as follows: 0 min 35% B, 1 min 35% B, 10 min 60% B, 10.5 min 99%B, 13min 99% B, 13.5 min 35% B, 17 min 35% B. Column effluent was introduced into the ESI source operated in negative ion mode as described by to Pedersen et al. [1] with some modifications: spray voltage -4500V; Temperature 550 C; CUR 35 L/min; GS1 60 L/min; GS2 50 L/min.

MRM transitions, retention time, internal standards and limit of detection are listed in Table S1. Additional parameters namely dwell time, collision energy (CE), collision exit potential (CXP) and declustering potential (DP) are listed in Table S2.

**Table S1.** MRM transitions for analyzed bile acids. BA standards are sorted by alphabetical order. RT: retention time (min), LOD: limit of detection (evaluated as S/N ratio 3:1).

| **Standard** | **Q1/Q3** | **RT (min)** | **LOD (ng/mL)** |
| --- | --- | --- | --- |
| 12-KCDCA | 405.3 / 405.3 | 5.42 | 0.25 |
| 7-KCDCA | 389.3 / 389.3 | 8.49 | 1.04 |
| 7-KDCA | 405.3 / 405.3 | 5.42 | 0.25 |
| CA | 407.3 / 407.3 | 7.09 | 1.11 |
| CDCA | 391.3 / 391.3 | 10.08 | 5.86 |
| CUDA | 339.3 / 214.3 | 9.25 | 16.3 |
| DCA | 391.3 / 391.3 | 10.46 | 1.09 |
| DHCA | 391.3 / 391.3 | 6.83 | 2.65 |
| DHLCA | 373.3 / 373.3 | 12.56 | 4.09 |
| GCA | 464.3 / 74.0 | 5.07 | 0.33 |
| GCDCA | 448.3 / 74.0 | 7.48 | 1.45 |
| GDCA | 448.3 / 74.0 | 7.97 | 0.68 |
| GHDCA | 448.3 / 74.0 | 5.06 | 1.82 |
| GLCA | 432.3 / 74.0 | 10.86 | 0.11 |
| GUDCA | 448.3 / 74.0 | 5.06 | 1.85 |
| HDCA | 391.3 / 391.3 | 7.49 | 4.90 |
| LCA | 375.3 / 375.3 | 12.62 | 14.9 |
| MCA | 391.3 / 391.3 | 6.83 | 2.65 |
| NDCA | 377.3 / 377.3 | 8.89 | 0.07 |
| TCA | 514.3 / 80.0 | 5.33 | 1.09 |
| TCDCA | 498.3 / 80.0 | 7.94 | 1.44 |
| TDCA | 498.3 / 80.0 | 8.56 | 0.73 |
| THCA | 449.3 / 449.3 | 11.03 | 50.1 |
| TLCA | 482.3 / 80.0 | 11.39 | 0.21 |
| TUDCA | 498.3 / 80.0 | 5.53 | 0.84 |
| T-α-MCA | 514.3 / 80.0 | 3.50 | 1.31 |
| T-β-MCA | 514.3 / 80.0 | 3.68 | 1.44 |
| T-ω-MCA | 514.3 / 80.0 | 4.48 | 5.16 |
| UCA | 407.3 / 407.3 | 4.04 | 1.65 |
| UDCA | 391.3 / 391.3 | 7.50 | 3.89 |
| α-MCA | 407.3 / 407.3 | 5.24 | 5.66 |
| β-MCA | 407.3 / 407.3 | 5.64 | 4.66 |
| γ-MCA | 407.3 / 407.3 | 6.39 | 0.54 |
| ω-MCA | 407.3 / 407.3 | 4.97 | 8.29 |

**Table S2.** Analyte-specific parameters used for MRM acquisition (Sciex 4000 QTRAP). CE: collision energy, CXP: collision exit potential, DP: declustering potential.

| **Name** | **Q1** | **Q3** | **Dwell (ms)** | **CE (V)** | **CXP** | **DP** |
| --- | --- | --- | --- | --- | --- | --- |
| 12-KCDCA\7-KCDCA | 405.3 | 405.3 | 50 | -30 | -9 | -130 |
| 7-KCDCA | 389.3 | 389.3 | 50 | -30 | -9 | -130 |
| UCA | 407.3 | 407.3 | 50 | -30 | -9 | -130 |
| CA | 407.3 | 407.3 | 50 | -30 | -9 | -125 |
| CA-D4 | 411.3 | 411.3 | 50 | -30 | -9 | -120 |
| CDCA\DCA | 391.3 | 391.3 | 50 | -30 | -9 | -130 |
| CDCA-D4 | 395.3 | 395.3 | 50 | -25 | -9 | -125 |
| CUDA | 339.3 | 214.3 | 50 | -35 | -3 | -65 |
| DCA-D4 | 395.3 | 395.3 | 50 | -30 | -9 | -125 |
| DHCA | 401.2 | 401.2 | 50 | -30 | -9 | -105 |
| DHCA\MCA | 391.3 | 391.3 | 50 | -30 | -9 | -105 |
| DHLCA | 373.3 | 373.3 | 50 | -30 | -9 | -130 |
| GCA | 464.3 | 74 | 50 | -70 | -4 | -125 |
| GCDCA-D4 | 452.3 | 74 | 50 | -65 | -4 | -120 |
| GDCA\GCDCA | 448.3 | 74 | 50 | -65 | -4 | -125 |
| GHDCA | 448.3 | 74 | 50 | -70 | -4 | -120 |
| GLCA | 432.3 | 74 | 50 | -65 | -4 | -120 |
| GUDCA | 448.3 | 74 | 50 | -70 | -4 | -115 |
| LCA | 375.3 | 375.3 | 50 | -35 | -8 | -130 |
| NDCA | 377.3 | 377.3 | 50 | -30 | -9 | -130 |
| TCA | 514.3 | 80 | 50 | -115 | -4 | -185 |
| TCDCA\TDCA\TUDCA | 498.3 | 80 | 50 | -110 | -4 | -145 |
| TCDCA-D4 | 502.3 | 80 | 50 | -110 | -4 | -175 |
| THCA | 449.3 | 449.3 | 50 | -30 | -10 | -140 |
| TLCA | 482.3 | 80 | 50 | -110 | -4 | -150 |
| T-α-MCA\T-β-MCA\ω-MCA | 514.3 | 80 | 50 | -110 | -4 | -155 |
| UDCA\HDCA | 391.3 | 391.3 | 50 | -30 | -9 | -125 |
| α-MCA\β-MCA\γ-MCA\ω-MCA | 407.3 | 407.3 | 50 | -30 | -9 | -115 |

**Table S3**. Primers used in this study

| Genes | Forward primer sequence 5’-3’ | Reverse primer sequence 5’-3’ |
| --- | --- | --- |
| Pol II | GACAACGAGGACAATTTCGACG | GGAGAATCTCGACATTTTCCTGG |
| CAR | CCCTGACAGACCCGGAGTTA | GCCGAGACTGTTGTTCCATAAT |
| PXR | GATGGAGGTCTTCAAATCTGCC | GGCCCTTCTGAAAAACCCCT |
| GR | AGCTCCCCCTGGTAGAGAC | GGTGAAGACGCAGAAACCTTG |

**Table S4.** Concentrations of XN and metabolites (IX, 8PN, DXN) in the plasma and liver of HFD-fed WT and *FXR^Liver-/-^* mice supplemented with 60mg XN/kg body weight/day. Data displayed as mean $\pm$ SEM (n = 15–17 per group). ^#^*p* < 0.05, ^##^*p* < 0.01 for genotype comparison. ND: not detectable

|  | Plasma (nM) | | Liver (nmol/g) | |
| --- | --- | --- | --- | --- |
|  | WT XN | *FXR^Liver-/-^* XN | WT XN | *FXR^Liver-/-^* XN |
| XN | 19.9 $\pm$ 2.8 | 23.0 $\pm$ 3.5 | 0.221 $\pm$ 0.03 | 0.219 $\pm$ 0.032 |
| IX | 12.6 $\pm$ 2.5 | 11.3 $\pm$ 1.9 | 1.135 $\pm$ 0.217 | 0.611^#^ $\pm$ 0.054 |
| DXN | 2.4 $\pm$ 0.6 | 2.4 $\pm$ 0.9 | *ND* | *ND* |
| 8PN | *ND* | *ND* | 0.048 $\pm$ 0.009 | 0.078 $\pm$ 0.023 |


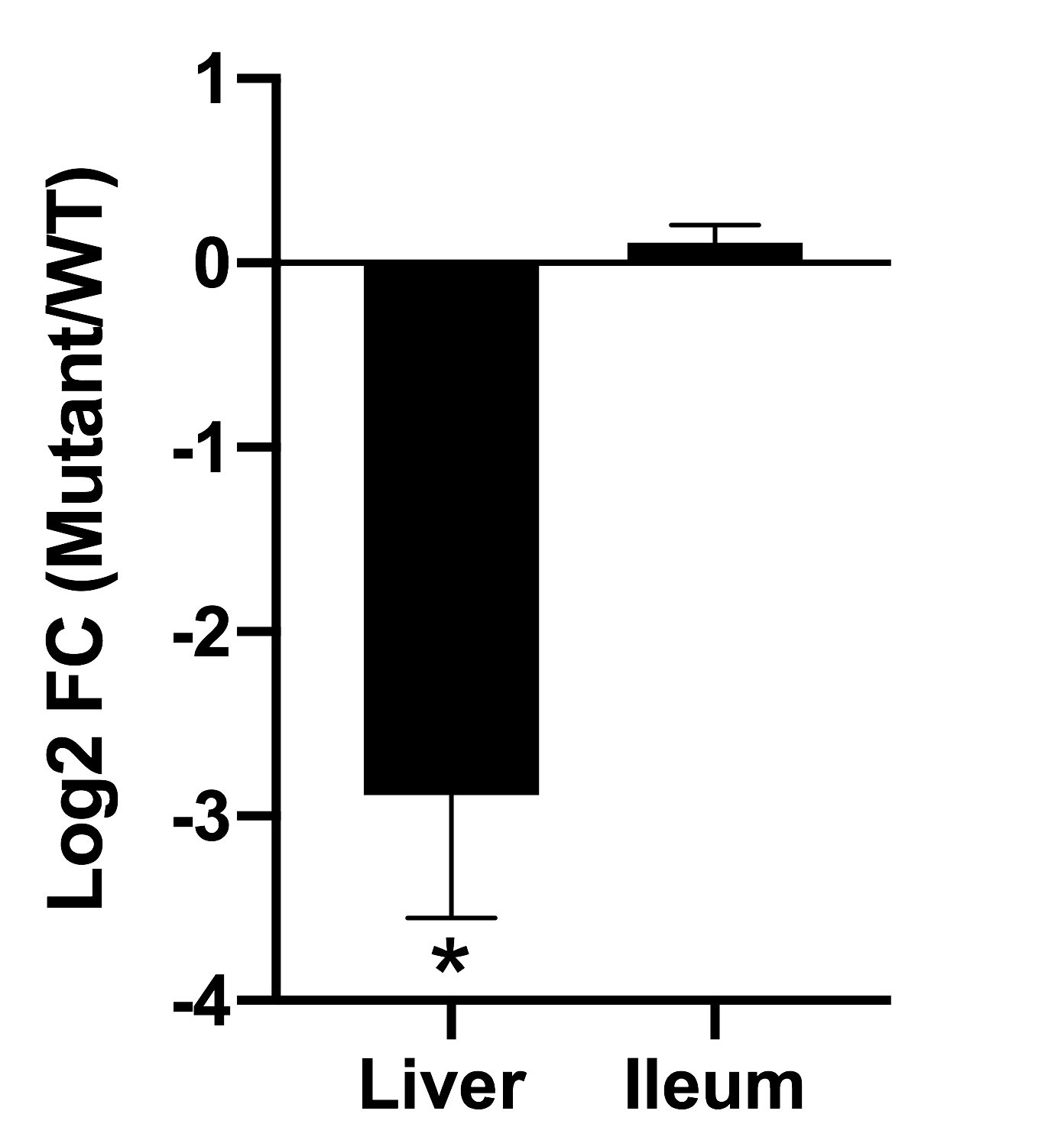


**Figure S1.** Real-time quantitative PCR analysis of FXR expression in the liver and ileum of *FXR^Liver-/-^* mice. Values are mean ± SEM (n = 4-5 per group), **p* < 0.05 for effect of phenotype.

**
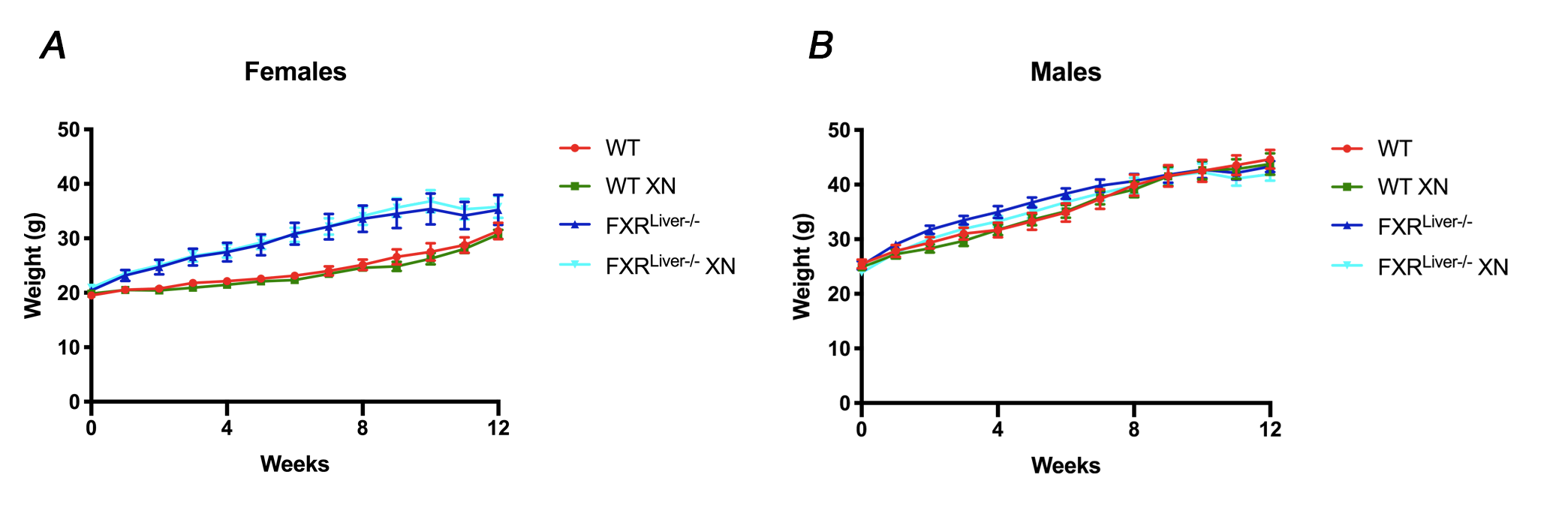
**

**Figure S2**. Body weight gain observed in HFD-fed (**A**) female and (**B**) male WT and *FXR^Liver-/-^* mice treated with XN. Values are expressed as mean ± SEM (n = 15-18 mice per group).


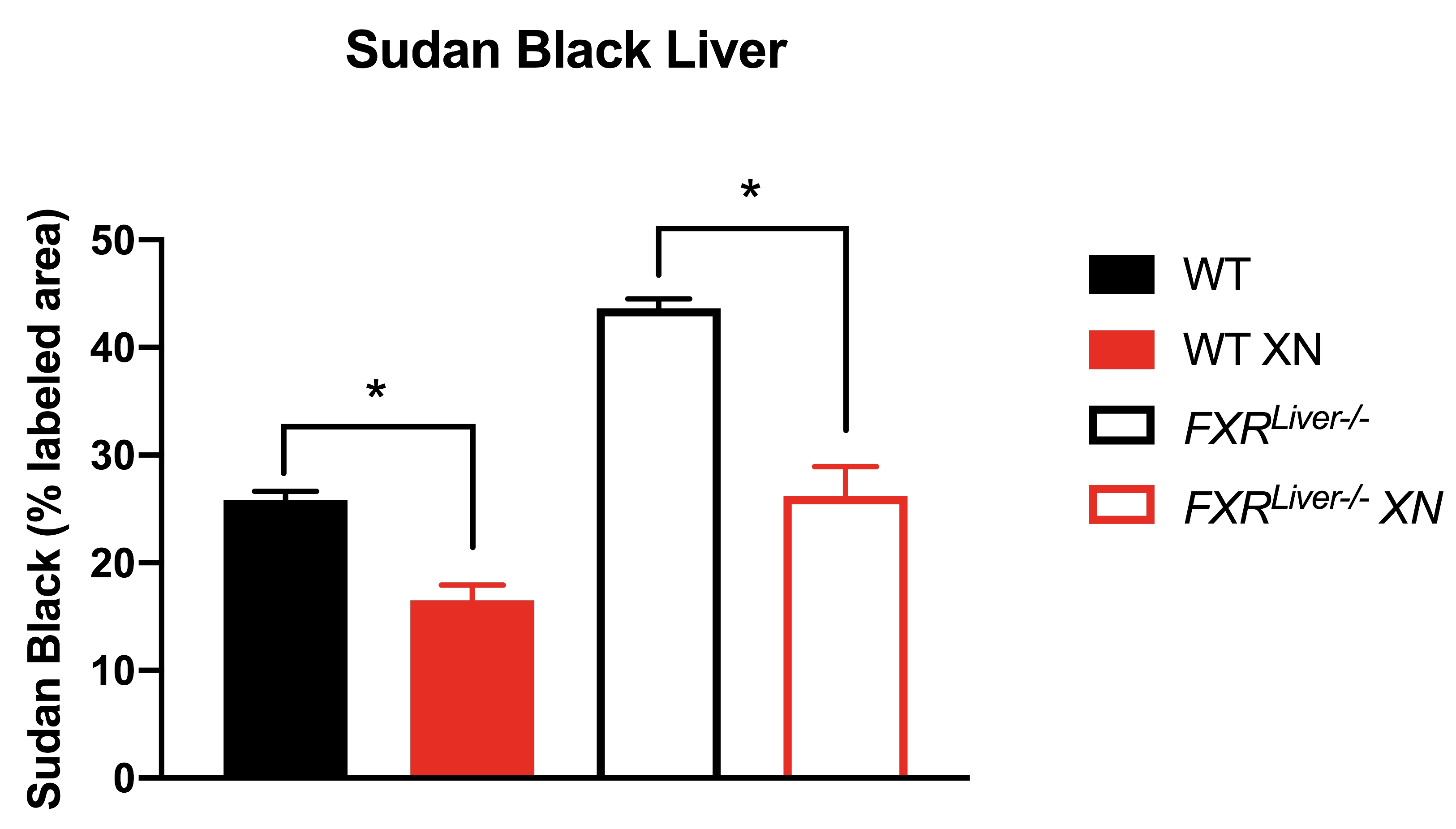


**Figure S3**. Percentage labeled area after Sudan Black staining of liver biopsies (n = 3 males per group) from HFD-fed WT and *FXR^Liver-/-^* mice treated with XN.


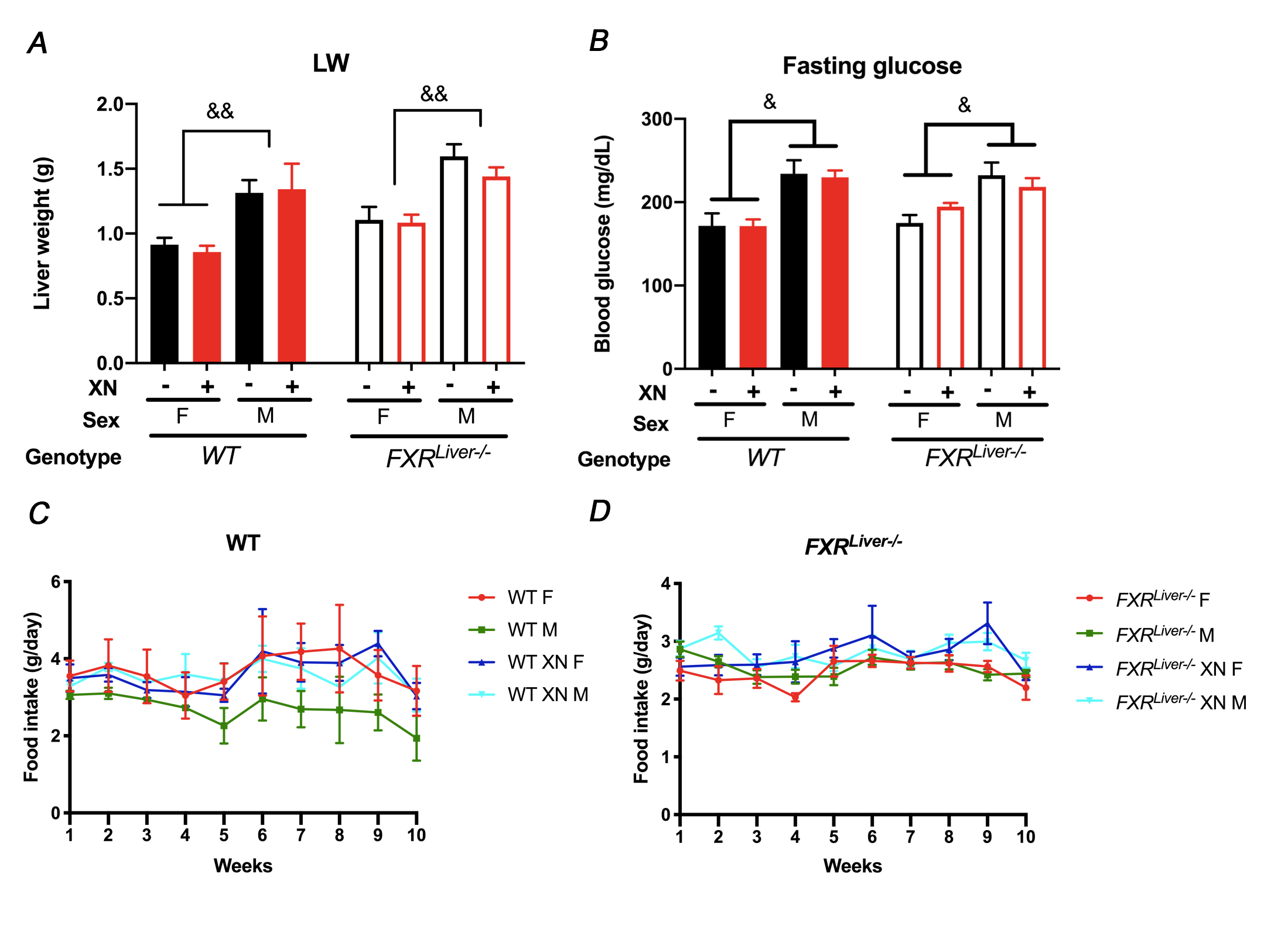


**Figure S4.** Liver weight **(A)** and fasting glucose **(B)** of HFD-fed WT and *FXR^Liver-/-^* mice untreated or treated with XN. Food intake of male and female WT **(C)** and *FXR^Liver-/-^* mice **(D)** over 10 weeks of the feeding experiment.


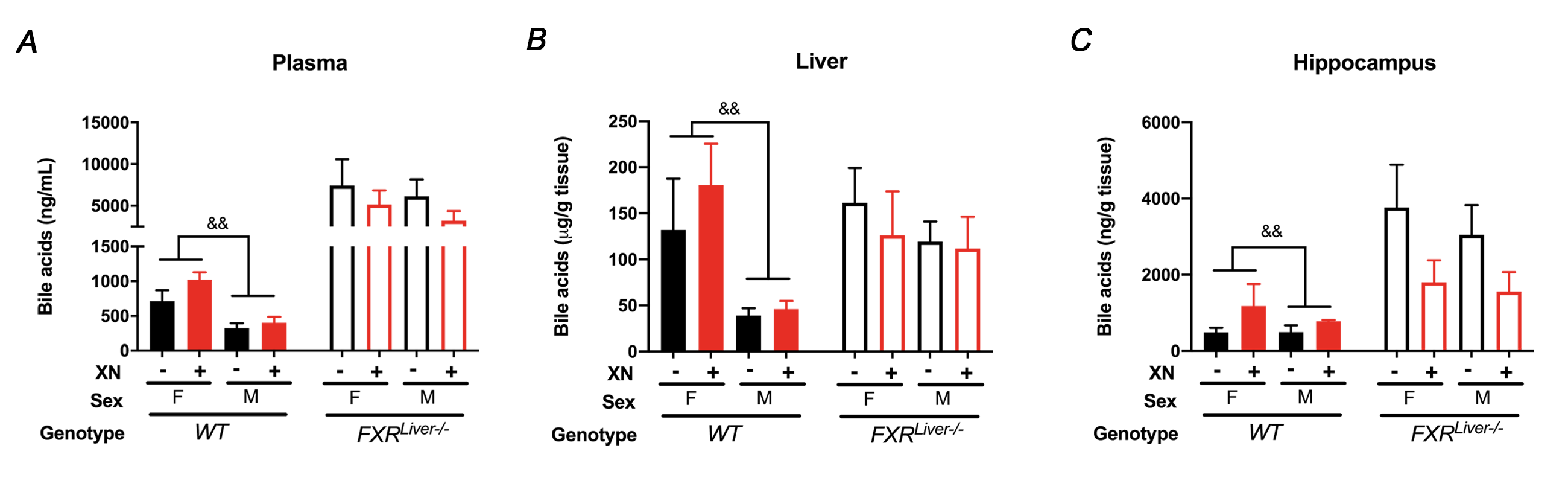


**Figure S5.** Effect of sex on bile acid concentrations in the **(A)** plasma, **(B)** liver and **(C)** hippocampus in male and female WT and *FXR^Liver-/-^* mice. Values are mean ± SEM (n = 7-10 per group). ^&^*p* < 0.05, ^&&^*p* < 0.01, ^&&&^*p* < 0.001 for gender comparison.


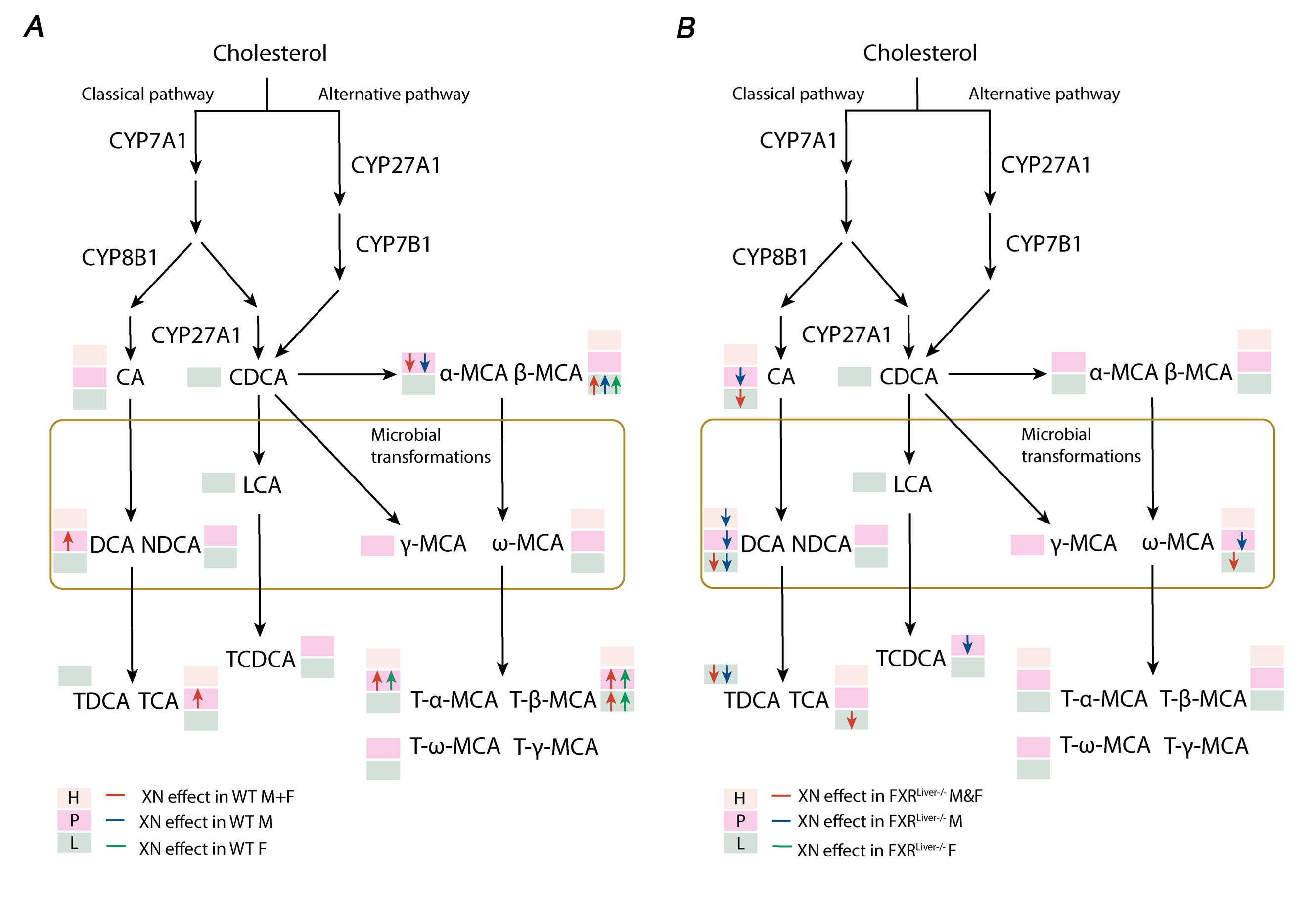


**Figure S6.** Effect of XN on the classical and the alternative pathway of BA synthesis in **(A)** WT and **(B)** *FXR^Liver-/-^* mice. Red arrows indicate significance in males and females combined, blue arrows indicate significance in males, green arrows indicate significance in females. Colored boxes indicate quantification of the BAs in the H (Hippocampus), P (Plasma) or L (Liver).

**Table S5.** Pathways (KEGG database) and number of genes affected in the liver of HFD-fed *FXR^Liver-/-^* mice.

| **Metabolic processes** | | **Inflammation and carcinogenic processes** | |
| --- | --- | --- | --- |
| Pathway | Hits | Pathway | Hits |
| Metabolic pathways | 129 | p53 signaling pathway | 17 |
| Glutathione metabolism | 18 | Cell cycle | 17 |
| Ferroptosis | 13 | ECM-receptor interaction | 13 |
| Metabolism of xenobiotics by cytochrome P450 | 15 | AGE-RAGE signaling pathway in diabetic complications | 14 |
| Drug metabolism - other enzymes | 18 | PI3K-Akt signaling pathway | 33 |
| Chemical carcinogenesis | 16 | FoxO signaling pathway | 16 |
| Drug metabolism - cytochrome P450 | 13 | Gap junction | 12 |
| Retinol metabolism | 14 | Focal adhesion | 21 |
| Carbon metabolism | 16 | Cellular senescence | 20 |
| Platinum drug resistance | 12 | MAPK signaling pathway | 26 |
| Fatty acid metabolism | 10 | EGFR tyrosine kinase inhibitor resistance | 10 |
| Protein digestion and absorption | 12 | HTLV-I infection | 22 |
| Fluid shear stress and atherosclerosis | 16 | Endometrial cancer | 8 |
| beta-Alanine metabolism | 6 | Proteoglycans in cancer | 19 |
| Biosynthesis of amino acids | 10 | Toxoplasmosis | 12 |
| Pentose and glucuronate interconversions | 6 | Thyroid cancer | 6 |
| Biotin metabolism | 2 | Tuberculosis | 17 |
| ABC transporters | 7 | Influenza A | 16 |
| PPAR signaling pathway | 10 | TNF signaling pathway | 12 |
| Amino sugar and nucleotide sugar metabolism | 7 | Melanoma | 9 |
| Fatty acid biosynthesis | 4 | Phagosome | 17 |
| 2-Oxocarboxylic acid metabolism | 4 | Th17 cell differentiation | 11 |
| Steroid hormone biosynthesis | 10 | Bladder cancer | 6 |
| Central carbon metabolism in cancer | 8 | Progesterone-mediated oocyte maturation | 10 |
| Porphyrin and chlorophyll metabolism | 6 | Antigen processing and presentation | 10 |
| Propanoate metabolism | 5 | Apoptosis | 13 |
| Pentose phosphate pathway | 5 | B cell receptor signaling pathway | 8 |
| Pyrimidine metabolism | 7 | Pathways in cancer | 38 |
| Sphingolipid metabolism | 6 | Complement and coagulation cascades | 9 |
| Glycerolipid metabolism | 7 | Colorectal cancer | 9 |
| Fatty acid degradation | 6 | Oocyte meiosis | 11 |
|  |  | Aldosterone-regulated sodium reabsorption | 5 |
|  |  | Rap1 signaling pathway | 17 |
|  |  | IL-17 signaling pathway | 9 |

References

[1] Pedersen, T. L., Newman, J. W., Establishing and Performing Targeted Multi-residue Analysis for Lipid Mediators and Fatty Acids in Small Clinical Plasma Samples. *Methods Mol Biol* 2018, *1730*, 175-212.
